# Multiplexed deactivated CRISPR-Cas9 gene expression perturbations deter bacterial adaptation by inducing negative epistasis

**DOI:** 10.1038/s42003-018-0135-2

**Published:** 2018-09-03

**Authors:** Peter B. Otoupal, William T. Cordell, Vismaya Bachu, Madeleine J. Sitton, Anushree Chatterjee

**Affiliations:** 1 Department of Chemical and Biological Engineering, University of Colorado at Boulder, Boulder, CO 80303 USA; 2 BioFrontiers Institute, University of Colorado at Boulder, Boulder, CO 80303 USA

## Abstract

The ever-increasing threat of multi-drug resistant bacteria, a shrinking antibiotic pipeline, and the innate ability of microorganisms to adapt necessitates long-term strategies to slow the evolution of antibiotic resistance. Here we develop an approach, dubbed Controlled Hindrance of Adaptation of OrganismS or CHAOS, involving induction of epistasis between gene perturbations to deter adaption. We construct a combinatorial library of multiplexed, deactivated CRISPR-Cas9 devices to systematically perturb gene expression in *Escherichia coli*. While individual perturbations improved fitness during antibiotic exposure, multiplexed perturbations caused large fitness loss in a significant epistatic fashion. Strains exhibiting epistasis adapted significantly more slowly over three to fourteen days, and loss in adaptive potential was shown to be sustainable. Finally, we show that multiplexed peptide nucleic acids increase the antibiotic susceptibility of clinically isolated Carbapenem-resistant *E. coli* in an epistatic fashion. Together, these results suggest a new therapeutic strategy for restricting the evolution of antibiotic resistance.

## Abstract (web summary)

Peter Otoupal et al. present CHAOS, an approach for preventing the development of antibiotic resistance in *E. coli* through CRISPR-Cas9-based perturbation of gene expression. They show that multiplexed perturbations decrease fitness of clinically-isolated Carbapenem-resistant *E. coli* upon antibiotic exposure.

## Introduction

The rapid emergence of multi-drug resistant superbugs poses a serious threat to millions and constitutes an impending international health crisis^1^. Bacteria are constantly driven to adapt to new treatments, establishing a biological arms race between evolution and our ability to develop new antimicrobial treatments. As the balance of these processes has trended towards more resistance and fewer novel therapies^2^, attention must be turned to new strategies which hinder the evolution of antibiotic resistance if the utility of our antibiotic arsenal is to be preserved. Current approaches are largely limited to the cycling of antibiotics, which rely upon high levels of coordination between clinicians and has been found to be difficult to implement in practice^3,4^. We propose an alternative strategy that we term Controlled Hindrance of Adaptation of OrganismS or CHAOS that involves introducing multiplexed gene expression perturbations to disrupt bacterial homeostasis by manipulating a fundamental process underpinning evolution: epistasis.

**Fig. 1:**
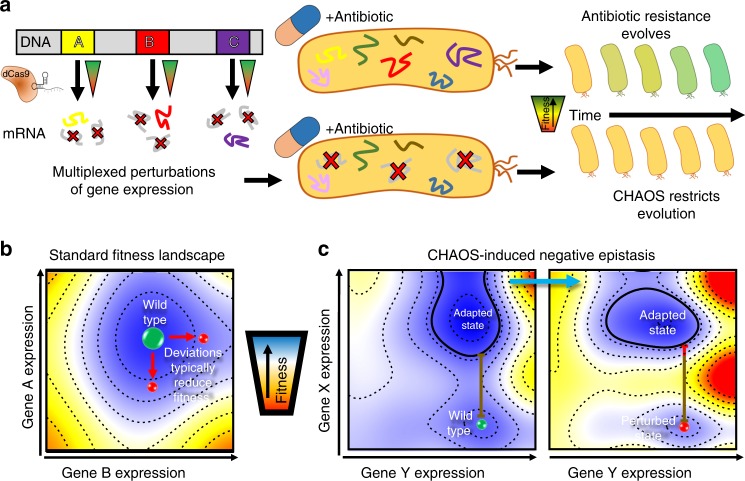
Controlled Hindrance of Adaptation of OrganismS (CHAOS) approach to deter evolution. **a** In CHAOS, basal levels of gene expression are perturbed in a targeted and multiplexed fashion. Introduction of these perturbations alongside an antibiotic disrupts the evolutionary forces driving the organism to evolve, thus restricting the increase in bacterial tolerance to said antibiotic over time. **b** The CHAOS approach is built upon the ideas used to explain how organisms evolve: fitness landscapes and epistasis. Here, a typical two-dimensional fitness landscape is presented based on various expression states of genes A and B (contours represent equal fitness). Although two genes are presented here, such a landscape can be constructed from any combination of an organism’s *n* × *n* genes. An underlying assumption of these landscapes is that epistasis has molded their shape such that deviations in gene expression from this basal state are typically detrimental to survival. **c** Wright’s shifting balance model of evolution^5^ predicts that in a small subset of these landscapes there exist adapted states where deviations in gene expression from basal wild-type levels improve fitness. However, these adapted states are separated by regions of fitness lower than the wild-type state that constrains the population. CHAOS can be employed to introduce multiplexed perturbations of gene expression that cause cascading epistatic effects, thereby reshaping the fitness landscape. This epistasis can be tuned to amplify the barrier to reach the adapted state, thus artificially constraining evolution

Epistasis describes the nonlinear outcome of combining two or more genetic changes. Negative (or positive) epistasis occurs if the combined changes produce a worse (or better) fitness than expected based upon their individual impacts. This phenomenon creates the rugged shapes of fitness landscapes used to describe evolution^5–7^. Such landscapes project an organism’s fitness at every unique genetic state for a pair of genes, with epistatic interactions influencing the landscape’s shape. Epistasis has been found to emerge frequently upon introduction of simultaneous mutations and is widely recognized to influence evolutionary trajectories^8–10^. Negative epistasis in particular has been shown to restrict rates at which bacteria adapt^11,12^. Research regarding epistasis has thus far focused on the theoretical side, using epistasis to explain how particular mutations emerged during evolution. Here, we demonstrate that artificial induction of epistasis has the potential for curtailing evolutionary adaptation by natural selection. This is accomplished by introducing multiplexed gene expression perturbations into a bacterium leading to cascading epistatic effects and restricting its ability to evolve (Fig. 1a).

Bacterial gene expression is stochastic in nature, allowing for heterogeneity throughout a genetically identical population. This provides an evolutionary advantage, in which some subpopulations with altered expression states can survive immediate exposure to a new and stressful environment^13–17^. However, such noise in gene expression comes at a fitness cost, as deviations from basal gene expression are often more deleterious than beneficial^18–21^ (Fig. 1b). This is strongly evidenced by tight expression of essential genes interacting with many genetic partners^22–24^. The high potential for deviations in these genes to trigger epistasis has been proposed as an evolutionary force constraining excessive heterogeneity^24^. We hypothesize that this could be exploited as a therapeutic strategy in which accumulating deviations in the expression of multiple genes would cause artificial induction of negative epistasis (Fig. 1c). As the shape of fitness landscapes used to describe evolution are directly related to the degree of epistasis, induction of epistasis can alter the adaptive trajectory available to a bacterium. Multiplexed perturbations could thus constrain an organism from reaching an adapted state, and offers a tangible avenue towards curbing the ability of bacteria to adapt to antibiotic treatment. Previous work in our lab investigating five strains harboring multiplexed gene expression perturbations^25,26^ showed their tendency to exhibit detrimental growth phenotypes^27,28^. Additionally, independently beneficial mutations of sequences related to gene expression were found to cause deleterious fitness impacts when combined jointly, further supporting the credibility of this hypothesis^29^.

Here we explore CHAOS-induced epistasis to restrict bacterial adaptation to antibiotics. We employ deactivated Cas9 (dCas9) clustered regularly interspaced short palindromic repeats (CRISPR) technology to selectively inhibit or activate mRNA production of specific genes. We construct a library of CRISPR plasmids to express dCas9 constructs for inducing gene expression perturbations and systematically quantify the impact of multiplexed gene perturbations on bacterial fitness with and without antibiotic exposure. We show that increasing the number of genes perturbed from their basal levels strongly correlates with reduced fitness during antibiotic exposure. We demonstrate that this phenomenon arises in a negative epistatic fashion, wherein compounding perturbations exacerbate fitness loss. The degree of negative epistasis correlated with the number of downstream genetic partners impacted by the primary gene perturbation(s). Disruption of genes relating to metabolic processes tended to induce the greatest level of negative epistasis. We demonstrate that negative epistasis correlated with significantly (*P* < 1 × 10^−16^) diminished adaptation rates over three to fourteen days and that such effects are sustainable, suggesting that multiplexed gene perturbations can be employed to restrict the evolution of antibiotic resistance. Finally, we use peptide nucleic acids (PNAs) to repress expression of a different set of gene perturbations in a multi-drug resistant clinical isolate of *E. coli*, demonstrating that CHAOS is applicable to other modes of gene expression manipulation. We successfully re-sensitized this antibiotic-resistant isolate to treatment, suggesting the benefit of CHAOS in clinical settings.

## Results

### Construction of CHAOS gene perturbation library

**Fig. 2:**
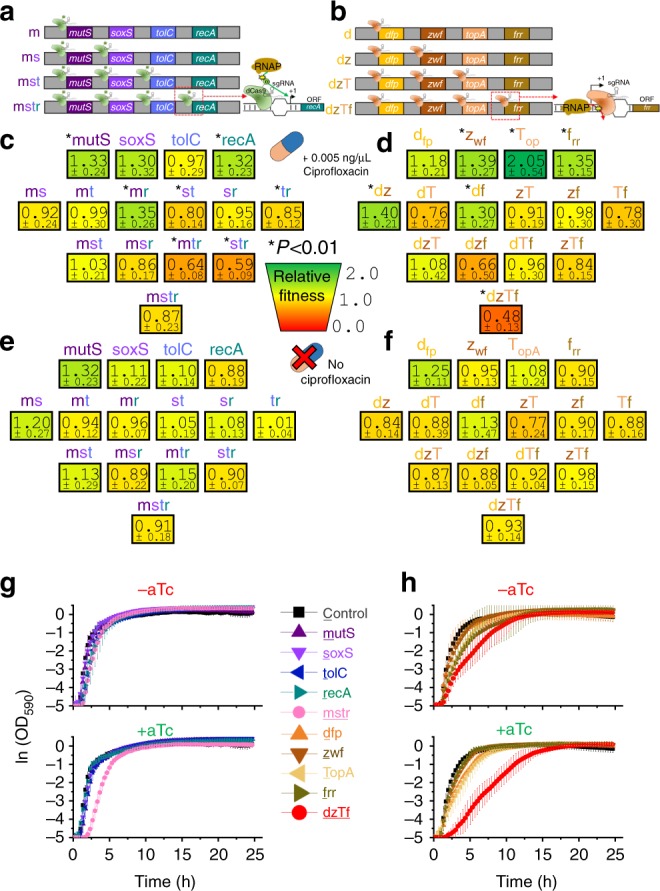
Negative fitness from multiple perturbations of gene expression. **a** Multiplexed CRISPR activation of stress response genes using aTc-inducible dCas9-ω engineered to bind upstream ~80–100 nt upstream of the +1 transcription start site. **b** Multiplexed CRISPR inhibition of conserved genes using aTc-inducible dCas9 engineered to bind overlapping the +1 transcription start site. **c**, **d** Fitness relative to control strain (*C*_mcherry_) after combinatorially increasing expression of stress response genes (**c**) or decreasing expression of conserved genes (**d**) during exposure to 0.005 µg mL^−1^ ciprofloxacin and 10 ng mL^−1^ aTc in LB medium. Relative fitness is listed below each strain name, followed by the standard deviation (*n* = 8). Asterisks to the left of strain names indicate significant fitness differences in relation to strain control grown under the same conditions and exhibiting a competitive fitness of 0.99 ± 0.07 (*P* *<* 0.01, two-tailed type II *t-*test). No significant differences were observed between strain control and another control strain CCCC harboring four nonsense gene perturbations and exhibiting a competitive fitness of 0.98 ± 0.14. **e**, **f** Fitness relative to control strain (C_mcherry_) from combinatorially increasing expression of stress response genes (**e**) or decreasing expression of conserved genes (**f**) during exposure to no ciprofloxacin and 10 ng mL^−1^ aTc in LB medium. Relative fitness is listed below each strain name, followed by the standard deviation (*n* = 3 for conserved gene strains, *n* = 4 for stress response gene strains). No strain was significantly different (at the *P* < 0.01 level) than the control strain CCCC with four nonsense gene perturbations grown in the same conditions and exhibiting average fitness of 0.93 ± 0.23 (*n* = 3, significance calculated using two-tailed type II *t*-test). **g**, **h** Growth of strains harboring stress response perturbations (**g**) or conserved gene perturbations (**h**) either individually or all at once during exposure to 10 ng mL^−1^ aTc in LB medium. Error bars represent standard deviation of five biological replicates

To explore artificial induction of negative epistasis, we constructed a library of *Escherichia coli* strains harboring dCas9^30^ or dCas9-ω^26^ devices to inhibit or activate mRNA production respectively. Each strain hosts a unique array of one or more single guide RNAs (sgRNAs) to direct the CRISPR enzymes to particular genomic loci (Fig. 2a, b).

We designed CHAOS strains targeting two gene sets. The first set included activation of four universal stress response genes important for adaptation^27,28^: *mutS* (DNA mismatch repair), *soxS* (SOX pathway regulator), *tolC* (multidrug efflux pump), and *recA* (SOS response activator) (Fig. 2a). These four genes were determined in our previous work to be particularly important during bacterial adaptation to a broad range of antibiotics and other stressors, and we reasoned that upregulating these genes would serve as the most likely avenue influencing adaptation^22,27,28^. Furthermore, we hypothesized that activating these genes would serve as the worst-case scenario, as we expect broad upregulation of stress response to provide multiple avenues of adaptive escape and encourage positive epistasis. The second set included inhibition of four genes found to be universally conserved across a broad set of bacteria central to distinct cellular pathways (Fig. 2b). We began with a set of 174 genes conserved across diverse bacterial genomes^31^, from which we selected genes that were not present in operons to minimize the number of genes directly perturbed by each CRISPR construct (Supplementary Table 1). From this, we selected well-characterized genes that were involved in diverse cellular processes in order to maximize potential epistatic effects. This led us to four promising conserved gene targets: *dfp* (synthesizes essential coenzyme A), *topA* (an essential supercoiling-relaxing enzyme), *zwf* (a key glycolysis enzyme), and *frr* (essential for ribosome recycling). These genes are monocistronic (except *tolC* and *recA*) and possess little direct interactions between one another (except *mutS/recA* and *soxS/tolC*) (Supplementary Figure 1).

We analyzed the STRING Database to determine the direct protein-protein interactions known for each gene and gene combination^32^. Multiplexing perturbation of conserved genes was predicted to cause more cascading impacts than multiplexing perturbation of stress response genes (Supplementary Figures 2–3). We predicted that perturbations within the conserved gene set would thus have a greater impact on fitness, as the cascading effects of the primary gene perturbation would affect more downstream partners in more diverse pathways.

We created CHAOS constructs with all possible single, double, triple and quadruple combinations of gene perturbations for each of these sets (Supplementary Figures 4–5 and Supplementary Table 2). A two-plasmid system was utilized to induce gene expression perturbation; the first plasmid encoded sgRNA target sequence(s), while the second plasmid encoded an anhydrotetracycline (aTc) inducible dCas9 or dCas9-ω for gene inhibition or activation respectively. Activation sgRNAs targeted ≈80–100 nt upstream of the +1 transcription start site of each gene^26^. Inhibition sgRNAs targeted the +1 transcription start site to inhibit transcriptional read-through via roadblock mechanism^30^. We engineered CRISPR perturbations to cause an approximately 10-fold range of under-expression or over-expression and verified the degree of perturbation using RT-qPCR (Supplementary Figure 6). The only unexpected result involved *topA* perturbation, which exhibited a likely Fis-dependent response (explored further in Supplementary Figure 6b).

We constructed two control strains targeting nonsense perturbation of the red fluorescent protein (*rfp*) gene, which was absent in all strains. These strains harbored one or four copies of *rfp* targeting sgRNAs (hereafter referred to as control and CCCC, respectively). Another control strain including constitutively expressed mCherry and one copy of *rfp* perturbation (*C*_mcherry_, see Methods) was created to enable tracking of the control population during strain competition. Finally, two control strains harboring either one or four sgRNAs to inhibit *lacZ* were constructed and used to demonstrate that no differences arose from targeting our control to a nonsense perturbation (*rfp*) or a gene irrelevant to fitness (β-*galactosidase* encoding *lacZ*) (Supplementary Figure 7). All subsequent experiments use nonsense *rfp* perturbations as controls.

### Combining gene perturbations reduces competitive fitness

We evaluated the fitness impacts caused by each CHAOS construct by competing these experimental strains with the fluorescent control strain *C*_mcherry_ during exposure to 0.005 µg mL^−1^ ciprofloxacin, a sub-minimal inhibitory concentration (MIC) allowing for moderate growth while still imparting selective pressure. We chose ciprofloxacin as it is a clinically-relevant antibiotic treatment which selects for resistant populations at very low concentrations^33^ via specific mutations in the *gyrA* gene^34^, thus allowing us to assess adaptation of strains. The CHAOS and controls strains were competed for one day followed by plating on solid media. Fitness impacts were quantified by measuring the relative changes in colony forming units of both control (*C*_mcherry_—red colonies), and the competed CHAOS strain before and after exposure to sub-MIC of ciprofloxacin using fluorescence imaging (see methods). No significant differences were observed between either control strains control or CCCC (exhibiting fitness of 0.99 ± 0.07 and 0.98 ± 0.14, respectively).

Individual gene perturbations of stress response genes either had no statistically significant impact (*soxS, tolC*) or increased fitness (*mutS, recA*) in relation to the control (Fig. 2c). A striking trend emerged as we multiplexed these four perturbations. While the average fitness of individual perturbations was improved (1.22 ± 0.30, *P* = 0.003), their benefits were abated when combined in pairs (0.98 ± 0.27, *P* = 0.88), triplets (0.78 ± 0.23, *P* = 0.002), and all at once (0.87 ± 0.23, *P* = 0.10) (Fig. 2c). Only one double perturbation was beneficial (*mutS-recA*), while another two (*soxS-tolC* and *tolC-recA*) resulted in significant fitness losses. Furthermore, half of the triple perturbations reduced fitness (*mutS-tolC-recA* and *soxS-tolC-recA*).

Perturbation of conserved genes resulted in a similar but even more drastic trend. Three of these perturbations significantly improved fitness (*zwf, topA*, and *frr*), with inhibition of *topA* providing a strong fitness benefit (Fig. 2d). As before, while the average fitness of these four perturbations was significantly improved (1.49 ± 0.44, *P* = 8 × 10^−5^), fitness decreased when these were combined in pairs (1.02 ± 0.34, *P* = 0.71) or three at a time (0.88 ± 0.38, *P* = 0.29). Combining all four perturbations resulted in a particularly severe diminishment of fitness (0.48 ± 0.13, *P* = 1 × 10^−11^), suggesting drastic epistatic effects (Fig. 2d). Collectively, these data support our hypothesis and demonstrate that fitness loss results from combinations of gene perturbations during antibiotic exposure.

We investigated if the inclusion of ciprofloxacin impacted these results by repeating the experiment without ciprofloxacin (Fig. 2e, f). The overall impact of perturbations was diminished under these conditions, and no strain exhibited a competitive fitness significantly different than the control (CCCC, 0.93 ± 0.23). These strains did exhibit an apparent trend towards lower fitness upon multiplexing; the average fitness of individual stress response gene perturbations (1.10 ± 0.24) decreased upon combining perturbation in pairs (1.04 ± 0.16), triplets (1.02 ± 0.23), and all at once (0.91 ± 0.18) (Fig. 2e). The same was true for multiplexing individual conserved gene perturbations (1.05 ± 0.20) into pairs (0.90 ± 0.27), triplets (0.91 ± 0.10), and all at once (0.93 ± 0.14) (Fig. 2f).

Growth curves of individual and four gene perturbations were also examined in the absence of ciprofloxacin (Fig. 2g, h). No growth changes were observed for any of the stress response gene perturbations in the presence or absence of aTc, while growth of strain mstr did diminish upon induction (Fig. 2g). Similarly, only minor impacts on growth emerged due to perturbation of conserved genes individually, while significant growth defects were present during induction of strain dzTf (Fig. 2h). All strains grew to similar maximum optical densities (ODs) by the end of 24 h. The growth impacts observed in the absence of aTc is likely due to leaky expression from the tet-promoter driving dCas9 and dCas9-ω expression. We quantified a ~100-fold increase in dCas9 expression upon aTc induction, with maximum induction occurring around 3.125 ng mL^−1^ (Supplementary Figure 8), less than the 10 ng mL^−1^ used in competition experiments.

Strain growth was analyzed in M9 minimal media to exacerbate potential growth impacts caused by gene perturbations. We varied concentrations from 0 to 50 ng mL^−1^ to parse the dCas9 response to induction. At high concentrations of aTc, we observed slight growth rate impacts on control strains, as would be expected for growth in minimal media with two antibiotics for maintaining two plasmids (Supplementary Figure 9). Again, no significant growth impacts were observed for any of the stress response gene perturbations (Supplementary Figure 10). Slight growth defects were observed due to conserved gene perturbations of *topA* at high aTc concentrations (Supplementary Figure 11). Perturbations of strains mstr and dzTf demonstrated significant growth defects at high aTc concentrations that were maintained up to the end 24 h of growth (Supplementary Figure 11). This resulted in significant reductions in growth rates at aTc concentrations used during competition (Supplementary Figure 12).

We also investigated whether growth in microplate cultures impacted our overall experimental outcomes, as growth in such conditions has frequently been correlated with oxidative stress due to poor oxygenation^35^. We found that batch growth competition resulted in similar conclusions as observed in microplate growth (Supplementary Figure 13).

Finally, we investigated whether inhibition or activation of gene expression had any impact on the phenomenon observed in Fig. 2c, d. For this, we created another four-perturbation strain activating expression of *mutS* and *soxS* while inhibiting expression of *topA* and *frr*. We tested the competitive fitness of this strain during ciprofloxacin exposure. While three of these perturbations significantly improved fitness, perturbation of all four simultaneously resulted in neutral fitness (1.09 ± 0.10) (Supplementary Figure 14). This strain also grew slower than the control strain, corroborating the notion that these perturbations interacted detrimentally upon multiplexing.

Taken together, these results demonstrate that individual gene perturbations were not detrimental to the fitness of *E. coli*. Only upon multiplexing these perturbations did significant growth and fitness impacts emerge, which were markedly more pronounced during exposure to ciprofloxacin. Thus, a clear trend towards lower fitness emerged upon multiplexing of gene perturbations.

### Negative epistasis emerges from combining gene perturbations

**Fig. 3:**
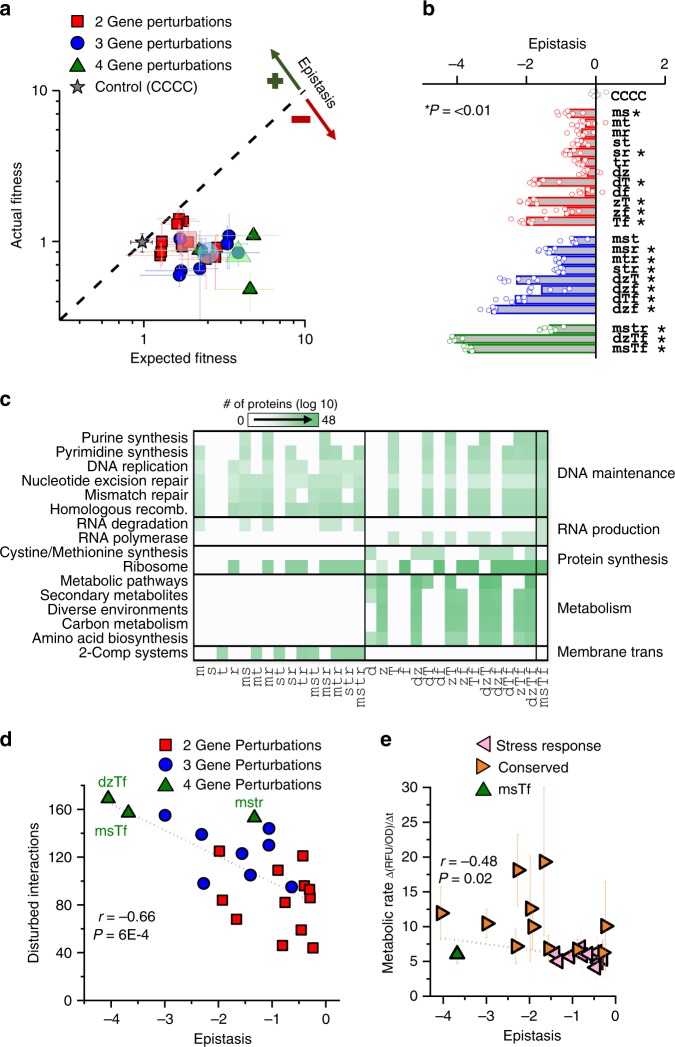
Epistasis resulting from two or more gene perturbations. **a** The relationship between expected and actual relative fitness of each strain harboring multiple gene perturbations. Centroid of each group (based on the number of genes perturbed) is shown by the larger transparent symbol. The dashed diagonal line indicates theoretical results if no epistasis was present. **b** Calculated epistasis of each strain (abbreviated as in Fig. 2). Error bars indicate standard deviation (*n* = 8). Raw expected fitness and epistasis values are presented in the Supplementary Data 1. Asterisks indicate significant negative epistasis in relation to the null hypothesis of zero epistasis (*P* *<* 0.01, Student’s *t-*test). **c** Counts of proteins directly impacted by gene perturbations, separated by functional classes as annotated by the Kyoto Encyclopedia of Genes and Genomes (KEGG). Only statistically significant (*P* *<* 0.05) over-represented KEGG pathways are reported. **d** Relationship between epistasis and the cumulative amount of direct protein interactions disturbed by each perturbation set. A linear fit is included, with Pearson correlation coefficient (r) and its significance (*P*). **e** The metabolic activity of all strains after sub-MIC ciprofloxacin exposure in LB medium quantified using fluorescence change from Resazurin dye. Fluorescence was normalized to final ODs (580 nm) of the respective replicate. Data represent the average of at least three biological replicates. A linear fit of the metabolic rate’s relationship to epistasis was performed using ANOVA associated r and *P* values presented

We next quantified epistasis between simultaneous perturbations by calculating deviations between the measured fitness of multiple perturbation strains, and their expected fitness based upon single perturbation (see methods). Comparing these fitness values, no deviation between expected and actual fitness of strain CCCC was observed, while multiple perturbation strains clearly exhibited lower fitness than was expected (Fig. 3a). This trend correlated into significant negative epistasis in half of the double perturbation strains and all but one of triple and quadruple gene perturbation strains (Fig. 3b). Notably, the only gene pairs known to interact (*mutS-recA* and *soxS-tolC*) did not demonstrate significant epistasis, indicating that direct interaction is not required to produce negative epistatic effects. The degree of negative epistasis also appeared to increase as more genes were perturbed. Inhibition of conserved genes resulted in statistically greater levels of negative epistasis than activation of stress response genes for triple perturbation constructs (*P* *=* 0.01). The high degree of negative epistasis across both sets appears compounded by sign-epistasis, wherein individually beneficial perturbations become deleterious once combined. Raw epistasis values and significance are presented in the Supplementary Data 1.

We investigated the functional processes influencing epistasis. We started by identifying all proteins known to directly interact with the gene(s) disrupted by CRISPR perturbation(s) as characterized by the STRING database^32^. Functional differences between the affected pathways from each set could explain these differences, as conserved gene perturbations disrupted pathways more central to cell survival. Exploring the functionality of each gene within the impacted networks, we observed that all simultaneous perturbation constructs impacted pathways related to DNA maintenance, RNA production, and protein synthesis, but metabolism and membrane transport were unique to conserved gene perturbations and stress response perturbations respectively (Fig. 3c). As expected, introducing more primary CRISPR perturbations introduced more cascading downstream impacts on protein partners within the *E. coli*. Interestingly, we observed a strong correlation (*P* *=* 6 × 10^−4^) between the total amount of affected partners and the degree of negative epistasis exhibited by each CHAOS strain (Fig. 3d). This suggests that as more interactions are disturbed from homeostasis, the greater the impact of epistasis on the cell’s overall health, likely due to increased disruption of homeostasis.

As central metabolic pathways were impacted only by conserved gene perturbations, we quantified metabolic rates of each strain after 20 h of exposure to sub-MIC levels of ciprofloxacin (Fig. 3e). We observed greater metabolic activity within the conserved set of perturbations (*P* *=* 8 × 10^−7^), and a significant correlation between metabolic activity and the degree of epistasis (*r* = −0.48, *P* *=* 0.02). A potential mechanism for this could be reduced efficacy of resource dedication towards surviving antibiotic exposure. This is supported by previous work correlating increased metabolic rates to potentiation of bactericidal antibiotics^36^ and another study correlating antibiotic efficacy to the presence of metabolites^37^. Furthermore, 13 of the affected metabolic genes in strain dzTf are essential. Adapting bacteria exhibit less stochastic expression in essential genes^22^, and negative epistasis has been proposed as a mechanism constraining this heterogeneity^24^ due to essential genes exhibiting stronger overall genetic interactions^38^. Indeed, genetic interactions are statistically greater in the conserved gene set than stress response set (Supplementary Figure 15, *P* *=* 7 × 10^−10^). Collectively, these studies appear to corroborate our finding that accumulating gene expression deviations in diverse cellular pathways has a fundamental tendency to influence fitness detrimentally in an epistatic fashion.

### Multiplexed perturbations slow bacterial adaptation rates

**Fig. 4:**
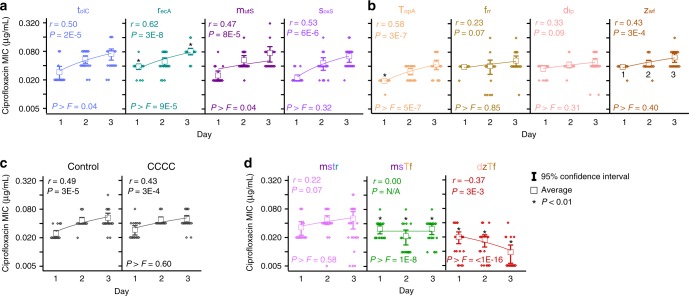
Perturbation of multiple genes slows bacterial adaptation. Change in the MIC of ciprofloxacin towards adapting populations at the end of each day of exposure for **a** strains harboring individual stress response gene perturbation constructs for *mutS*, *soxS*, *tolC*, and *recA* activation, **b** strains harboring individual conserved gene perturbation constructs for *dfp*, *zwf*, *topA*, and *frr* inhibition, **c** control strains, and **d** strains with simultaneous perturbation of four genes corresponding to stress response genes only (mstr), combination of stress response and conserved genes (msTf), and conserved genes only (dzTf). Each box-plot (*n* = 22, individual data points shown) includes a linear fit with associated Pearson correlation coefficient (*r*) and its significance (*P*). *F*-tests were performed for strain’s linear fit against the linear fit of strain control during the same experiment, and the resulting significance is reported as *P* > *F*. Raw MIC values are presented in the Supplementary Data 1. Asterisks indicate significantly different average MICs in relation to strain control during the same day and experimental run (*P* *<* 0.01, two-tailed type II *t-*test)

To test the hypothesis that induced negative epistasis can restrict the adaptive potential of bacteria, we exposed single and quadruple perturbation strains to ciprofloxacin over three days (D1–3) and quantified changes in MIC (Fig. 4, see methods). The trajectory of MIC change was quantified using Pearson correlation coefficients of linear fits over time (*r*, see methods). Statistical differences in fits between the control and CHAOS strains were estimated using *F*-tests (reported as *P* > *F* values).

Most single perturbation strains and the controls adapted similarly, with MICs increasing over time. *recA* activation resulted in a significant increase in MIC on D1 (*P* *=* 2 × 10^−5^) and D3 (*P* *=* 2 × 10^−3^), and the rate of this increase was significantly faster than the control (*P* *>* *F* = 9 × 10^−5^) (Fig. 4a). While *topA* perturbation lowered MIC on D1 (*P* *=* 5 × 10^−15^), the strain quickly adjusted back to control levels resulting in a statistically faster rate of increase than the control (*P* *>* *F* = 5 × 10^−7^) (Fig. 4b). The discrepancy between fitness and initial MIC impacts of individual *topA* perturbation may be due to the aforementioned gene expression dependency on cell phase. As expected, no differences were observed between single and four gene control perturbation strains (Fig. 4c).

Experimental strains of quadruple perturbations exhibited striking differences in adaptive trends. Strain mstr (*mutS*, *soxS*, *tolC*, and *recA*) exhibited a positive correlation that was weaker than each individual perturbation (Fig. 4d). Strain msTf (*mutS*, *soxS*, *topA*, and *frr*) exhibited a completely flat MIC trajectory, and always survived statistically lower ciprofloxacin concentrations than the control. Strain dzTf (*dfp*, *zwf*, *topA*, and *frr*) presented the most striking results, as not only were MICs statistically lower than the control, average MIC actually decreased over time (*r* = −0.37, *P* *=* 3 × 10^−3^). This was primarily due to death of fifteen replicates by the end of adaptation. Nevertheless, excluding replicates that died during the course of the experiment showed a neutral trajectory of MIC over time (*r* = −0.09, *P* *=* 0.56), indicating that the living population was adapting slower (Supplementary Figures 16–17). The failure of strain dzTf to adapt to ciprofloxacin exposure was reproducible across multiple experimental runs (*r* = −0.42, *P* *>* *F* *=* 7 × 10^−15^) (Supplementary Figure 18). Strains exhibiting greater negative epistasis (dzTf and msTf) also adapted at slower rates, suggesting a correlation between the degree of epistasis and the rate of adaptation. Raw MIC values for all strains discussed above are presented in the Supplementary Data 1. These results demonstrate the potential of employing CHAOS to introduce epistasis into an organism and constrain rates of bacterial adaptation to antibiotics over time.

To determine if differences in MICs translated to changes at the genetic level, we sequenced each strain at the end of three days of ciprofloxacin exposure. We focused specifically on *gyrA*, as mutations in this gene (S83L and D87Y) have been found as the first genetic changes during the evolution of ciprofloxacin resistance^34^. The vast majority of strains exhibited no *gyrA* mutations (Supplementary Table 3). In total, only four isolates exhibited any *gyrA* mutations. These included one replicate of the control, and one replicate each of strains with individual perturbation of *mutS*, *soxS*, and *topA*. Increased perturbations did not appear to bias cells to mutate more, as no replicates of mstr, msTf, and dzTf exhibited mutations in *gyrA*. Mutation fluctuation assays corroborate this, revealing similar mutation rates across all strains with the exception of individual *mutS* and *soxS* perturbation (Supplementary Figure 19). Due to similar mutation rates, differences in adaptive trajectories across strains appear to be driven primarily by changes in gene expression.

**Fig. 5:**
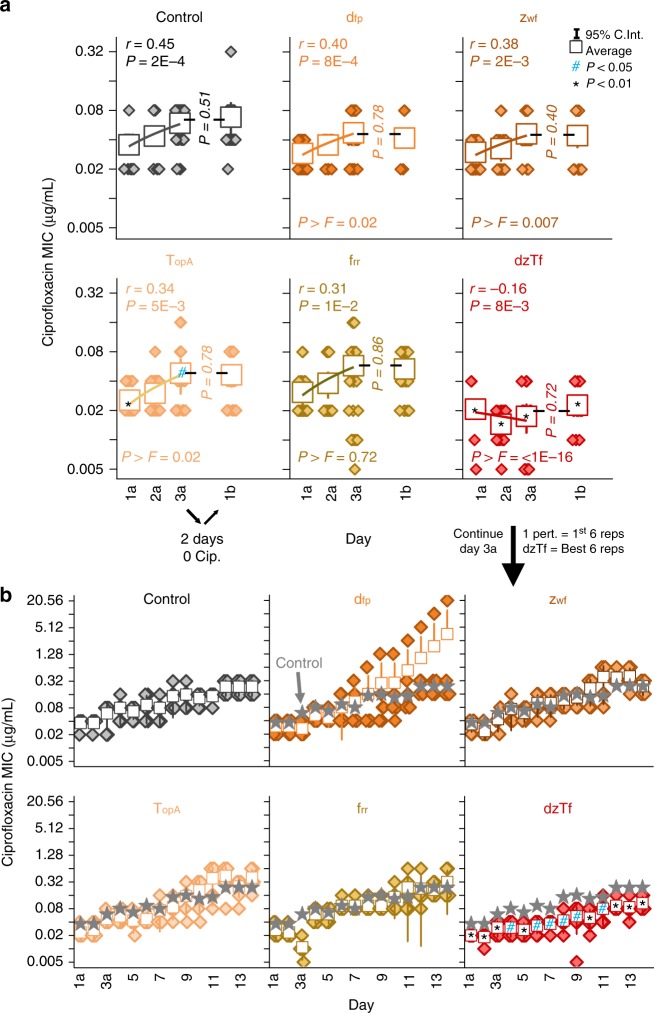
The impact of multiplexed conserved gene perturbation is maintained in the absence of ciprofloxacin and for longer periods of adaptation. **a** Change in the MIC of ciprofloxacin towards adapting populations of strains harboring the noted perturbation constructs Each box-plot (*n* = 22, individual data points shown) includes a three-day linear fit with associated Pearson correlation coefficient (*r*) and its significance (*P*). *F*-tests were performed for strain’s linear fit against the linear fit of strain control during the same experiment, and the resulting significance is reported as *P* > *F*. At the end of the third day (3a), samples were inoculated into LB with induction and grown for two days in the absence of ciprofloxacin. The MICs of these samples were measured again after one day (1b) of ciprofloxacin exposure. **b** The experiment was continued for six replicates of each strain at the end of three days of exposure (3a) for day four to day 14. The first six replicates for all strains besides dzTf, which continued the best six replicates reaching the highest MICs. Stars are overlain on each graph to show the average MIC of the control strain. All asterisks (*) and hashtags (#) indicate statistical difference from the control strain on the same day of the experiment (*P* *<* 0.01, two-tailed type II *t-*test)

We further explored strain adaptability by looking at the sustainability of MIC changes. We again adapted each of the individual conserved gene perturbations, as well as strains dzTf and the control, for three days (Fig. 5a). We found similar results as before, with strain dzTf adapting particularly poorly. After three days of exposure, all strains were removed from ciprofloxacin exposure for two days to reset the phenotypic state, after which they were re-exposed to ciprofloxacin gradients. Despite growth in the absence of ciprofloxacin for two days, every strain survived to the same levels of ciprofloxacin it had on day three. This suggests that the bacteria exhibited sustainable adaptive changes in response to ciprofloxacin, except for the bacteria exposed to multiplexed perturbations inducing negative epistasis.

Finally, we also examined the adaptive potential of these strains in a longer, clinical relevant timeframe of two weeks (Fig. 5b). Six replicates of each of the six strains from the above experiment were taken at the end of day three, and continued for another 11 days of adaptation to ciprofloxacin. We chose the first six replicates of each of the individually perturbed strains to continue with, and biased our experiment by picking the six best replicates of strain dzTf to continue with adaptation. This was done to see if a subset of the dzTf population was able to escape the epistatic effect and evolve resistance to ciprofloxacin. Despite this bias, strain dzTf never managed to reach similar levels as the control strain throughout the entire experiment. The population did begin surviving higher ciprofloxacin levels around day eight of the experiment, suggesting that adaptation was eventually possible. But the speed at which this strain evolved resistance was clearly diminished, suggesting that CHAOS restriction of adaptation is applicable in clinically relevant timeframes.

### PNA perturbations increase susceptibility of MDR *E. coli*

To demonstrate the therapeutic potential of CHAOS we multiplexed gene perturbations against a clinical isolate of carbapenem-resistant Enterobacteriaceae (CRE) *E. coli*, a bacterial pathogen recently designated as priority 1 critical class by the World Health Organization^39^. Characterization of this isolate using the 2016–2017 Clinical & Laboratory Standards Institute (CLSI) sensitive/resistant breakpoint values^40^ showed resistance to at least eight classes of antibiotics^41,42^. For this study, we tested the response of this strain to chloramphenicol as it exhibited greater than 8-fold higher MIC (>256 µg mL^−1^) than the corresponding CLSI breakpoint of 32 µg mL^−1^ (Supplementary Table 4). We targeted four new genes, allowing us to confirm if CHAOS is generalizable. Based on the success of CHAOS strain dzTf, we chose to target essential genes representing diverse cellular pathways. These genes included *folC*, an H2-folate synthetase involved in folate biosynthesis^43^, *ffh* encoding a signal recognition particle protein gene essential for protein translocation^44^, *gyrB* encoding gyrase subunit B important for transcription, and an essential noncoding small RNA *fnrS*.

**Fig. 6:**
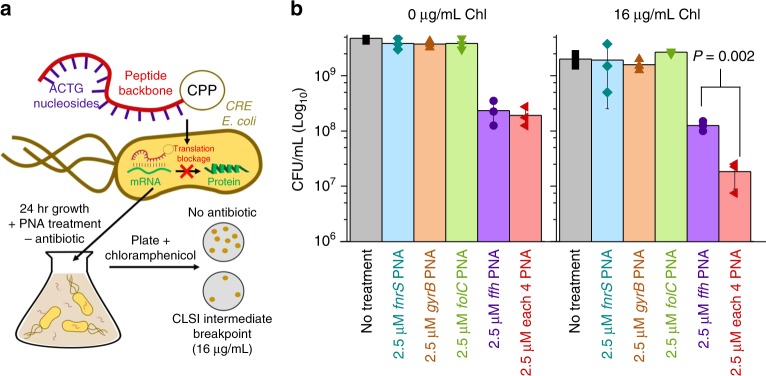
CHAOS increases the antibiotic susceptibility of clinically isolated CRE *E. coli*. A CRE isolate of *E. coli* exhibiting resistance to at least 11 antibiotics above CLSI breakpoint levels was isolated from a clinical infection (Supplementary Table 4). We focused on applying CHAOS induced epistasis to re-sensitize this isolate to chloramphenicol. **a** A new set of four universally conserved bacterial genes were perturbed using PNA to demonstrate applicability outside of CRISPR interference and towards clinically relevant infections. PNA structure consists of a peptide backbone connecting nucleosides analogous to DNA and linked to a cell penetrating peptide. These molecules are able to enter bacteria and anneal tightly to analogous mRNA sequences, allowing for targeted blockage of protein translation. Chloramphenicol-resistant CRE *E. coli* was exposed to 2.5 µM of four unique PNAs either individually or in combination (for a total concentration of 10 µM PNA) for 24 h, after which cells were plated on both plain LB agar, as well as clinically-relevant levels of chloramphenicol to determine viable cells. **b** CFU analysis of CRE *E. coli* after exposure to PNA treatment demonstrates CHAOS’s effectiveness. Exposure to PNA *ffh* resulted in a ~16-fold reduction in viable cells with respect to no PNA treatment, while the remaining PNAs exhibited largely no effect under both conditions. Combination of all 4 PNAs exacerbated chloramphenicol’s toxicity and gave rise to ~110-fold reduction in viable cells with respect to no PNA treatment in an apparently epistatic fashion even at sub-resistance levels. *P* values were calculated using two-tailed type II *t*-test

As delivery of CRISPR systems into bacteria is still difficult to achieve therapeutically, we employed an alternative gene expression repression strategy based on 12-mer peptide nucleic acids (PNAs, abbreviated as α-*gene*). These PNAs target and bind to the translation start codon of these genes, thus inhibiting translation of these genes’ mRNAs into protein^45^ (see Methods). We chose concentrations of PNA at 2.5 µM at which individual perturbations had minimal to no effect on cell growth of standard MG1655 *E. coli*. These PNAs were conjugated via an O-linker to a cell-penetrating peptide (CPP) motif for direct intracellular delivery. We exposed the clinically isolated CRE *E. coli* to these PNAs individually and in combination for 24 h, before plating in both the presence and absence of chloramphenicol (Fig. 6a).

We observed that in absence of chloramphenicol, the total amount of viable cells of the isolate was affected only by α-*ffh* (16-fold decrease with respect to no PNA treatment), with no apparent exacerbation of α-*ffh*’s toxic effect upon multiplexing with other PNAs (Fig. 6b). When these same cultures were plated in the presence of 16 µg mL^−1^ of chloramphenicol, an intermediate CLSI-breakpoint level, similar results were observed for all of the individual PNA treatments. However, multiplexing of all four PNAs significantly reduced the viability of the clinical isolate by nearly 110-fold with respect to no PNA treatment and resulted in a significant reduction (*P* = 0.002) compared to the best individual perturbation. We note that overall PNA concentration was increased under this condition, leading to the potential of slightly increased antibiotic permeability (Supplementary Figure 20). However, such impacts were relatively small, and disappeared after 15 h of growth. Taken together, this data provides evidence that CHAOS can be used to increase the susceptibility of highly drug-resistant clinical isolates in an apparently epistatic fashion.

## Discussion

This work provides the framework for CHAOS, a new strategy to slow bacteria’s ability to adapt to antibiotics by employing multiplexed perturbations of gene expression to induce negative epistasis. The influence of gene expression epistasis on evolutionary trends is a relatively recent and virtually unexplored idea. The first study to suggest epistasis might exist on the gene expression level (rather than the mutational level) emerged in 2013^24^, and 2014 saw the first representation of fitness landscapes based on gene expression^29^. While the exact role of gene expression epistasis in influencing evolution is a subject of debate^46^, the data presented here supports the notion that excessive divergence from basal expression in multiple genes induces negative epistasis. Surprisingly, this effect appears even when no direct genetic interactions exist. This effect appears to be influenced by the number of perturbations, as well as the type of pathways affected (Fig. 3).

An intuitive explanation for this phenomenon is that increasing perturbations generally leads to a breakdown in homeostasis, as shifts in independent pathways create cascading detrimental impacts. For example, despite no direct interaction between *topA* and *zwf*, both plausibly influence DNA folding rates through different mechanisms—*topA* through altered topoisomerase expression, and *zwf* through altered NADPH production, influencing flux through the thioredoxin pathway that regulates *gyrA* expression^47^. Such collateral effects help explain why independent perturbation of *topA* and *zwf* appears to increase fitness during ciprofloxacin exposure, but their combined perturbation results in negative epistasis. Furthermore, this disruption of homeostasis via gene expression changes could explain why essential genes are conspicuously restricted in heterogeneity^24^, particularly after stress exposure^22^.

Our previous results also corroborate this notion, wherein a smaller set of five strains harboring multiplexed inhibitions and activations of two to three stress response genes tended to exhibit negative epistasis when exposed to various stressors including both antibiotics and biofuels^27^. This raised the idea that gene expression based epistasis might be a fundamental phenomenon, paving the way for this work in which we significantly expand this idea.

Artificially engineering bacterial gene expression to increase antibiotic susceptibility is a relatively new idea. Previous work has shown multiplexing transcription factor overexpression can successfully re-sensitize antibiotic resistant bacteria^48^, but is limited to mass alterations of genetically related genes regulated by the same native processes bacteria have naturally evolved. In contrast, CHAOS can theoretically be applied to modify any set of genes and can be interfaced with either a CRISPR or PNA platform.

One of the major limitations to implementing CHAOS in a clinical setting is the delivery of these gene perturbation systems into the target cells, especially for intracellular infections. Significant work is being undertaken to improve the therapeutic delivery of CRISPR systems^49–54^ and PNAs^55–57^, and there is reasonable potential that these approaches could be applied to making CHAOS a viable therapy. This is best demonstrated by our application of CHAOS to increase the susceptibility of a clinically isolated multi-drug resistant CRE *E. coli*. Despite high levels of resistance, we were able to increase its susceptibility to chloramphenicol with multiplexed perturbations of four essential genes.

Hindering the evolution of antibiotic resistance is a major goal of antibiotic synergy in combination therapy approaches^58^, and engineered negative epistasis affords an ancillary but functionally separate strategy to achieve this goal. Exploiting genetic interactions is ripe for use in therapeutic strategies, as evidenced by the growing interest in synthetically lethal mutations that are detrimental to cancer mutated cells but benign to healthy cells^59^. Future studies involving a systematic investigation of transcriptome-wide epistasis would help in this regard by identifying optimal gene combinations to achieve detrimental interactions with minimal invasiveness. Such studies will help to confirm whether the sign of perturbation (activation vs. inhibition) or the degree of perturbation could influence the level of emergent epistasis. Overall, our results demonstrate that applying a more sophisticated understanding of gene expression enables one to induce negative epistasis to gain control over adaptation rates, and could preserve the efficacy of last-resort antibiotics when employed in co-therapy strategies.

## Methods

### Plasmid and strain construction

A list of plasmids and primers used in this study can be found in Supplementary Tables 2 and 5 respectively. All plasmids have also been deposited to Addgene with plasmid numbers 114,632–114,665. A two-plasmid system was utilized to induce native gene expression perturbation; the first plasmid encoded a sgRNA target sequence, while the second encoded either dCas9 or dCas9-ω for gene inhibition and activation respectively. Addgene plasmid 44,249 was used directly for targeting *rfp* inhibition (the Control—C) and served as the starting plasmid for creating all subsequent sgRNA plasmids. Addgene plasmid 44,251 was used directly for providing dCas9, while the previously constructed pPO-dCas9ω^27^ plasmid was used directly for providing dCas9-ω. New sgRNA target plasmids were created by replacing the RFP-targeting sequence in 44,251 with new gene sequences specific to the target of interest. This was accomplished by designing unique forward primers flanked with a *SpeI* restriction site and encoding the new target sequence. A common reverse primer flanked with *ApaI* was used alongside these primers to perform PCR amplification with Phusion High-Fidelity DNA Polymerase (New England Biolabs) of DNA inserts, which were subsequently digested with Cutsmart SpeI and ApaI (New England Biolabs) alongside 44,251 backbone. Ligations of these pieces were performed using T4 DNA Ligase (Thermo Scientific), which were subsequently transformed into electrocompetent NEB 10-β. Transformants were minipreped using Zyppy Plasmid Miniprep Kit (Zymo Research Corporation) and submitted for sequencing to confirm successful insertion (GENEWIZ). sgRNA plasmids targeting individual genes were used to construct sgRNA plasmids targeting two or more genes via Gibson Assembly, for which a common forward and reverse primer was used to amplify the first sgRNA target plasmid while introducing overhangs downstream of the terminator sequence following the first target. A common set of primers were then utilized to amplify sgRNA targets to the second, third, and fourth targets depending on the intended number of final sgRNA targets. A batch Gibson reaction was performed at 50 °C for 3 h with T5 exonuclease (New England Biolabs), Phusion polymerase and Taq ligase (New England Biolabs) on this one backbone and one to three inserts to stitch all pieces together (Supplementary Figure 4). sgRNA-C-mCherry was constructed by amplifying constitutively expressed mCherry from pFPV-mCherry (Addgene 20,956) and inserting into 44,251 upstream of the *rfp* sgRNA sequence. Final experimental sgRNA plasmids were transformed into chemically competent *E. coli* strain K-12 MG1655 (ATCC 700926) harboring either 44,249 or pPO-dCas9ω if the target was meant to inhibit or activate expression respectively. This process was used to construct all control and experimental strains used in the study.

### Media and culture conditions

All cultures were grown in Lennox Luria-Bertani Broth (LB) (Sigma-Aldrich), with the exception of RT-qPCR samples and certain samples for growth calculations which were grown in M9 minimal media (5× M9 minimal media salts solution from MP Biomedicals, 2.0 mM MgSO_4_, and 0.1 mM CaCl_2_ supplemented with 0.4% weight/vol glucose). Plates and media were supplemented with ampicillin (100 µg mL^−1^) or chloramphenicol (35 µg mL^−1^) to maintain selection of sgRNA plasmids or dCas9/dCas9-ω plasmids respectively. aTc was used to induce CRISPR expression at a final concentration of 10 ng mL^−1^, except where otherwise noted. The authors also note that the aTc-inducible promoter driving expression of dCas9 is not P_L_tetO-1 as originally reported^30^, but rather a tet-promoter variant with only one Tet binding site highly similar to the original tet-promoter, indicating that slightly higher leaky expression is expected of dCas9 and dCas9-ω. All cultures were grown at 37 °C, with shaking at 225 rpm. Cultures for competition were grown in 200 µL cultures in 96-well conical-bottom microplates. Cultures for RT-qPCR and for batch growth comparisons to microplates were grown in 3 mL cultures. Cultures for CFU and MIC screens were grown in 100 µL cultures in 384 well flat-bottom microplates. Cultures for growth rate calculations were grown in 100 µL cultures in 384 well flat-bottom microplates in a GENios plate reader (Tecan Group Ltd.) operating under Magellan software (version 7.2) with 16.6 min of shaking before measurement of optical densities at 590 nm absorbance every 20 min.

### Quantitative reverse transcription PCR

The degree of gene expression perturbation was confirmed by subjecting biological triplicates of each individual gene perturbation to RT-qPCR, as well as constructs perturbing four genes simultaneously and the control strain targeting *rfp*. Cultures were inoculated from individual colonies and grown for 20 h overnight in 3 mL M9 cultures and subsequently diluted 1:100 the following morning into 3 mL of fresh media containing aTc. These cultures were grown for 8 h before RNA extraction using the GeneJET RNA Purification Kit (Thermo Scientific) and purification using the Turbo DNA-free kit (Ambion). Separate biological replicates of *topA* perturbation and the single target *rfp* control strain were also grown for 24 h to stationary phase and collected as before. Purified RNA was used to create cDNA using the Maxima First Strand cDNA Synthesis Kit for RT-qPCR (Thermo Scientific). Technical duplicates of each replicate were subjected to RT-qPCR reactions from the Maxima SYBR Green qPCR Master Mix (Thermo Scientific) using 2 ng of cDNA in 12.5 µL reactions run on a QuantStudio 6 Flex Real-Time PCR System (Applied Biosystems) in the CU Core Sequencing Facility. Reactions were allowed to run for 40 cycles with Rox normalization. Gene expression changes were calculated using 2^-ΔΔCq^ values calculated from averages of technical duplicates, relative to the control strain targeting *rfp*.

### Competition fitness assay

The fitness of each perturbed strain was calculated by competing said strain against the red fluorescent control strain C_mCherry_. A total of eight biological replicates were inoculated from colonies into 200 µL in 96 well plates and grown overnight for 16 h with selection. Cultures were then diluted 1:100 into 200 µL of media with selection and 10 ng mL^-1^ aTc to induce gene perturbation and grown for another 24 h. Competition was initiated by diluting cultures 1:100 and mixing equal cell ratios of the red control strain with each experimental strain into 200 µL of media containing selection, 10 ng mL^−1^ aTc and 0.005 µg mL^−1^ ciprofloxacin. To determine starting ratios of each strain, two µL were diluted into 198 µL of water, from which 10-fold serial dilutions up to 1:100,000 were created each using a total volume of 200 µL for each dilution. 50 µL of both the 1:10,000 and 1:100,000 dilutions were plated to determine CFUs (and by extension starting ratios) of each strain. The remaining 198 µL of culture not used for serial dilutions was grown for another 24 h, diluted 1:100 into the 198 µL of the same media, and grown again for 24 h. At the end of this growth period, CFUs for each strain were determined using the previously outlined dilution scheme. Plates were incubated for 24 h after inoculation. Two images were taken of each plate with fluorescence activation at 540 nm, one with emission filtering at 590 nm and the other without. These images were overlaid to facilitate colony counting. Colony counts were used to determine fitness values (**ω**) using the standard Malthusian fitness equation:^60^

$$\omega = \ln \left( {N_{E1} \times 100^2/N_{E0}} \right)/\ln \left( {N_{C1} \times 100^2/N_{C0}} \right),$$

where the variables are defined as follows: **N**: CFU, **E**: experimental strain, **C**: control strain, **1**: after exposure, and **0**: before exposure. Fitness values were calculated as such for all experimental strains, as well as the control strains targeting *rfp* with one or four sgRNAs and expressing no fluorescence. This same protocol was repeated in the absence of ciprofloxacin for all combinations of conserved gene perturbations

### Epistasis calculations

Expected fitness values (**ω**_**e**_) for strains with perturbation of two or more genes were calculated assuming a multiplicative model as follows:

$${\boldsymbol{\omega }}_{\boldsymbol{e}} = \mathop {\prod }\limits_{i = 1}^n {\boldsymbol{\omega }}_{\boldsymbol{i}},$$

where *n* expands to all sets of genes perturbed. For instance, **ω**_**e**_ of strain dzf would be calculated as the product of fitness from each individual gene perturbation (*ω*_*d*_ × *ω*_*z*_ × *ω*_*f*_). Epistasis (**E**) was calculated as the difference between measured fitness and expected fitness (*E* = *ω* − *ω*_*e*_).

### Growth assay

To demonstrate growth phenotypes, biological triplicates of each strain were inoculated from individual colonies into 150 µL of LB containing selection in a conical 96 well microplate and grown for seven hours. After initial growth, one µL of each culture was used to inoculate two mL of M9 or LB media containing selection and grown for 16 h. The following morning, each culture was diluted 1:100 into 100 µL M9 or LB media cultures containing selection and a variable concentration of aTc from 0 to 50 ng mL^−1^. These cultures were grown in 96 or 384 well microplates in a Tecan GENios reader for 24 h, measuring Optical Density at 590 nm every 20 min.

### Resazurin metabolic rate assay

Four individual colonies were cultured overnight (16 h) in 100 µL of LB supplemented 35 µg mL^−1^ of chloramphenicol, and 100 µg mL^−1^ ampicillin a 384 well microplate. These cultures were then diluted 1:100 into LB supplemented with cm, amp, 0.005 µg mL^−1^ ciprofloxacin, and 10 ng mL^−1^ aTc. The microplate was then incubated in a Tecan GENios microplate reader with continuous shaking at 37 °C while measuring optical densities (580 nm). After 20 h of growth, 10x Resazurin (a dye which fluoresces brightly upon interaction with intracellular NADH, thereby quantifying metabolic activity) was added to each well (final concentration of 0.01 mg mL^−1^), and fluorescence (excitation 485 nm and emission 610 nm) was measured in 5-minute intervals. The slope of the curve from the first five data points was used to quantify metabolic activity during the initial stages of ciprofloxacin exposure.

### Minimum inhibitory concentration assays

MIC assays were performed using 22 biological replicates per strain. Individual colonies were inoculated into 100 µL LB cultures containing selection and grown for 16 h overnight. The following morning, cultures were diluted 1:50 into 100 µL of fresh media containing 10 ng mL^−1^ aTc in 384 well plates and grown another 24 h. The following day, each replicate was diluted 1:50 into fresh media containing selection, aTc, and a range of ciprofloxacin concentrations including 0, 0.005, 0.01, 0.02, 0.04, 0.08, and 0.16 µg mL^−1^ ciprofloxacin to begin the MIC screen. The new 384 well plate containing variable ciprofloxacin concentrations was grown for 24 h (Day 0–Day 1), after which absorbance was measured at 590 nm. Cultures expressing ODs greater than 0.20 were determined to have survived. The highest concentration at which each replicate survived was used to inoculate the same plate setup as defined previously (Day 1–Day 2), while the next highest concentration was determined to be the MIC. This process was repeated for one more day to obtain MICs for 22 cultures at the end of each day of growth for all three days. Three replicates of each strain growing at the highest MICs were saved as glycerol stocks for subsequent sequencing.

Samples for re-exposure to ciprofloxacin were diluted 1:50 into fresh media containing 10 ng mL^−1^ aTc, chloramphenicol, ampicillin and no ciprofloxacin. These cultures were grown for 24 h, diluted into fresh media, and grown for another 24 h. After two days of growth in the absence of ciprofloxacin, these cultures were transferred back into the ciprofloxacin concentration gradient using the aforementioned protocol.

The same batch of 22 cultures used for re-exposure to ciprofloxacin experiments was used for the fourteen-day adaptation experiment. For this, the first six replicates by order of the control strain, as well as individual perturbations of *dfp*, *zwf*, *topA*, and *frr* were continued on for up to 14 days using the same protocol as before, but increasing the maximum concentration of the gradient up to 20.48 µg mL^−1^ ciprofloxacin. For strain dzTf, we biased our experiment by picking the six best replicates as quantified by maximum MIC reached.

### Mutation sequencing assay

Glycerol stocks of strains saved after three days of ciprofloxacin exposure were streaked onto LB agar plates with selection and grown overnight. Two colonies from each plate were used to perform colony PCR amplification of *gyrA* in the 1203 bp region surrounding S83 and D87, the most likely regions for mutations conferring ciprofloxacin resistance to arise. PCR samples were purified and submitted for sequencing (GENEWIZ), for a total of six samples per strain.

### Mutation fluctuation assay

Mutation rates were estimated using the rifampicin exposure approach outlined by Luria and Delbruck^61^. Individual colonies were grown in three mL LB without selection for 16 h and subsequently adjusted to normalized ODs with the addition of LB to denser cultures. Each culture was used to dilute 1:10,000 into 33 parallel 100 µL cultures of LB supplemented with 10 ng mL^−1^ aTc and grown for 24 h. Colony forming units were estimated from three replicates on plain LB agar plates, while the remaining 30 cultures were plated on LB agar containing 100 µg mL^−1^ rifampicin. Colonies were counted after 48 h of exposure, and the FALCOR web tool was used to estimate mutation rates^62^.

### Statistical analyses

Unless otherwise indicated, all *P* values reported were calculated using a standard two-tailed type II student’s *t*-test. Pearson correlation coefficients and their corresponding *P* values were calculated using linear fits with no weighting (OriginPro 9.3.226 software). Comparisons of linear fits (*P* *>* *F* values) were performed using *F*-tests of results from linear fits in OriginPro. Grubb’s test for outliers was used to remove individual data points prior to calculations where indicated. Standard deviations of expected fitness (*σ*_*e*_) of simultaneous gene perturbations were calculated from standard deviations of single gene perturbations (σ_*i*_) using error propagation following the equation: ${\mathrm{\sigma }}_e = {\mathrm{\omega }}_e \times \sqrt {\mathop {\sum }\limits_{i = 1}^n \left( {\frac{{\sigma _i}}{{\omega _i}}} \right)^2}$. Standard deviations of epistasis (σ_*E*_) were also estimated using error propagation following the equation: ${\mathrm{\sigma }}_E = \sqrt {\sigma ^2 + \sigma _e^2}$ where σ is the standard deviation of measured fitness of the combined gene perturbation strain. We determined whether epistasis values deviated from the null hypothesis (zero epistasis) by performing a one-sample *t*-test to obtain *P*-values (assuming a two-tailed distribution) using the standard formula $t = \left( {E - \mu } \right)/\left( {{\mathrm{\sigma }}_E/\sqrt n } \right)$ where the variables are defined as follows: *t* is the test statistic, *E* is the average sample epistasis, *μ* is the null hypothesis (in this case, zero), σ_*E*_ is the estimated standard deviation of Epistasis, and *n* is the number of replicates (in this case, eight).

### Peptide nucleic acid (PNA) design and synthesis

PNA polymers, peptide backbones connecting standard ATCG nucleosides which bind strongly to RNA and act as effective translation inhibitors, were purchased pre-synthesized (PNA Bio Inc.). These polymers were bought conjugated to a cell-penetrating-peptide motif (KFF)_3_K connected to the C-terminus by an O-linker and supplied as a lyophilized powder. Upon arrival, PNA was resuspended in 5% DMSO to a final concentration of 100 µM. PNA sequences were designed to bind to 12 nts surrounding the translation start site of the targeted gene, keeping minimal off-target homology. These PNA sequences from N to C terminus are as follows: (KFF)_3_K-O-ATACCATGATTAT (*folC*), (KFF)_3_K-O-CTCTTGCAGGTG (*fnrS*), (KFF)_3_K-O-GACAATGTTTGA (*ffh*), and (KFF)_3_K-O-GTTGATGTCGAA (*gyrB*). The nonsense PNA targeting a non-existent RNA sequence was designed using the sequence GAATAAGGGCGA and also included the (KFF)_3_K motif on the N terminus. This PNA was graciously donated to us from Dr. Teruna Siahaan of the University of Kansas.

### Multiplexed PNA treatment of CRE *E. coli*

Carbapenem-resistant Enterobacteriaceae (CRE) *E. coli* was obtained resistance levels was obtained from the lab of Dr. Nancy Madinger at the University of Colorado Anschutz Medical Campus, and its resistance profile was characterized in previous work^41,42^. For testing CHAOS against this strain, the clinical isolate was plated on cation-adjusted Muller Hinton Broth (caMHB). Three individual colonies were picked from this plate and grown in 3 mL caMHB overnight for 16 h. Each culture was then diluted 1:100 into a new 50 µL cultures of fresh caMHB in a 384 well plate and grown for another 24 h. Each culture was then diluted 1:100 again into six new 50 µL cultures of fresh caMHB in a 384 well plate, this time supplemented with either no PNA, 2.5 µM of each PNA individually, or 2.5 µM of each PNA mixed together (for a final concentration of 10 µM PNA). These cultures were then grown for another 24 h before performing CFU analysis on plates supplemented with either no chloramphenicol or supplemented with 16 µg mL^−1^ chloramphenicol. Plates were incubated for 24 h after plating before performing CFU analysis.

A control experiment testing the effect of increased PNA concentration was also run using the control nonsense targeting PNA. Individual colonies of the CRE *E. coli* were picked and used to inoculate three 3 mL caMHB overnight for 16 h. Cultures were then diluted 1:100 into fresh 50 µL caMHB supplemented with either 0, 2.5, or 10 µM of the nonsense PNA in both the presence and absence of 16 µg mL^-1^ chloramphenicol in a 384 well microplate. These cultures were then grown in a microplate for 24 h with continuous shaking while tracking ODs.

## Electronic supplementary material

Supplementary Information

Description of Additional Supplementary Files

Supplementary Data 1

## Data Availability

All data generated or analyzed during this study are included in this published article (and its supplementary information files). Raw excel files of all data are available from the corresponding author or first author on reasonable request.
