# Supplementary material for: Multiplexed deactivated CRISPR-Cas9 gene expression perturbations deter bacterial adaptation by inducing negative epistasis
Source: Commun Biol. 2018 Sep 3;1:129. doi: 10.1038/s42003-018-0135-2 (PMC6123780; doi:10.1038/s42003-018-0135-2)
Supplement: Supplementary file 1 — Supplementary Information [file 42003_2018_135_MOESM1_ESM.pdf]

Supporting Online Material for

Multiplexed deactivated CRISPR-Cas9 gene expression perturbations deter bacterial adaptation by inducing negative epistasis

Peter B. Otoupal, William T. Cordell, Vismaya Bachu, Madeleine J. Sitton and Anushree Chatterjee[*].

*To whom correspondence should be addressed.
E-mail: Chatterjee@Colorado.EDU

This PDF file includes:

Supplementary Figures 1-20
Supplementary Tables 1-5
Supplementary References 1-2

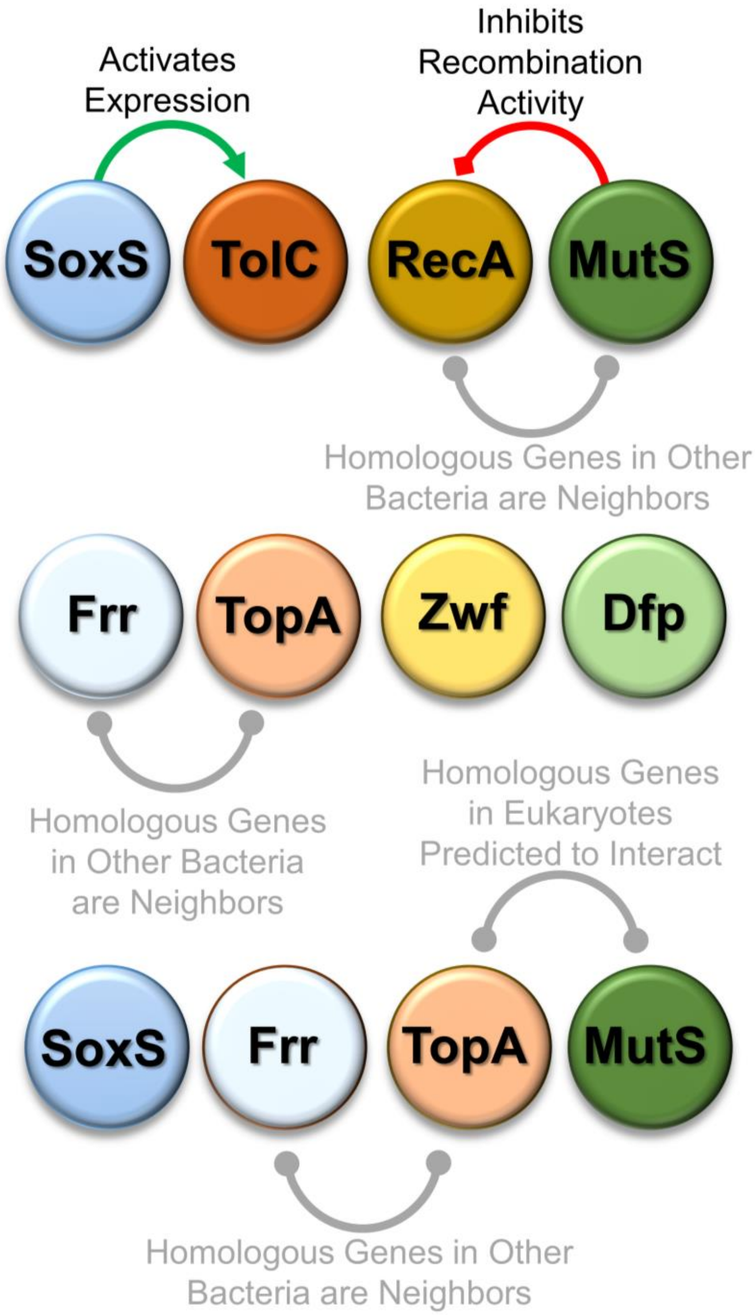

Supplementary Figure 1. All known genetic interactions of gene targets investigated in this study. Possible uncharacterized interactions are also listed in gray, based on homology to other organisms. Interactions were determined from the STRING protein database[1].

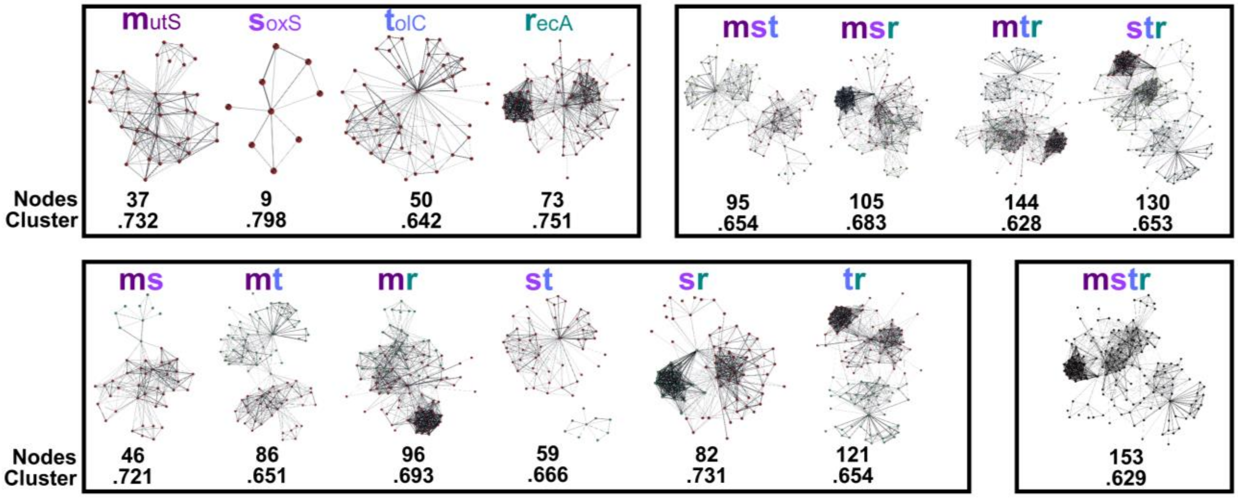

Supplementary Figure 2. Predicted networks of proteins impacted by each gene perturbation within the stress response set, including all combinations of perturbations affecting *mutS*, *soxS*, *tolC*, and *recA* abbreviated as in Figure 2. All known potential primary protein partners were determined from the STRING protein database[1] using information from databases, co-expression, co-occurrence, gene fusion, and experimental validation. A minimum interaction score confidence level of 0.4 was used. Each node represents a unique protein, with lines between each node representing a predicted interaction. Line thickness represents the predicted confidence of protein-protein interaction. Below each network is listed the number of nodes contained within, as well as the cluster coefficient. Cluster coefficients predict the overall interconnectedness of the network, with higher values indicating a "tighter" network of more closely associated interactions.

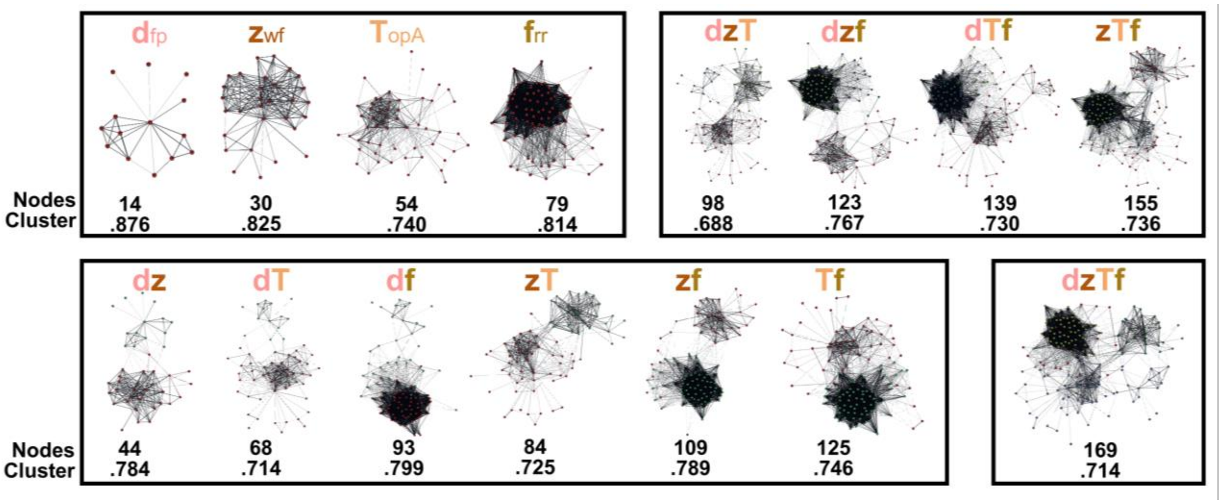

Supplementary Figure 3. Predicted networks of proteins impacted by each gene perturbation within the conserved set, including all combinations of perturbations affecting *dfp*, *zwf*, *topA* and *frr* abbreviated as in Figure 2. All known potential primary protein partners were determined from the STRING protein database[1] using information from databases, co-expression, co-occurrence, gene fusion, and experimental validation. A minimum interaction score confidence level of 0.4 was used. Each node represents a unique protein, with lines between each node representing a predicted interaction. Line thickness represents the predicted confidence of protein-protein interaction. Below each network is listed the number of nodes contained within, as well as the cluster coefficient. Cluster coefficients predict the overall interconnectedness of the network, with higher values indicating a “tighter” network of more closely associated interactions.

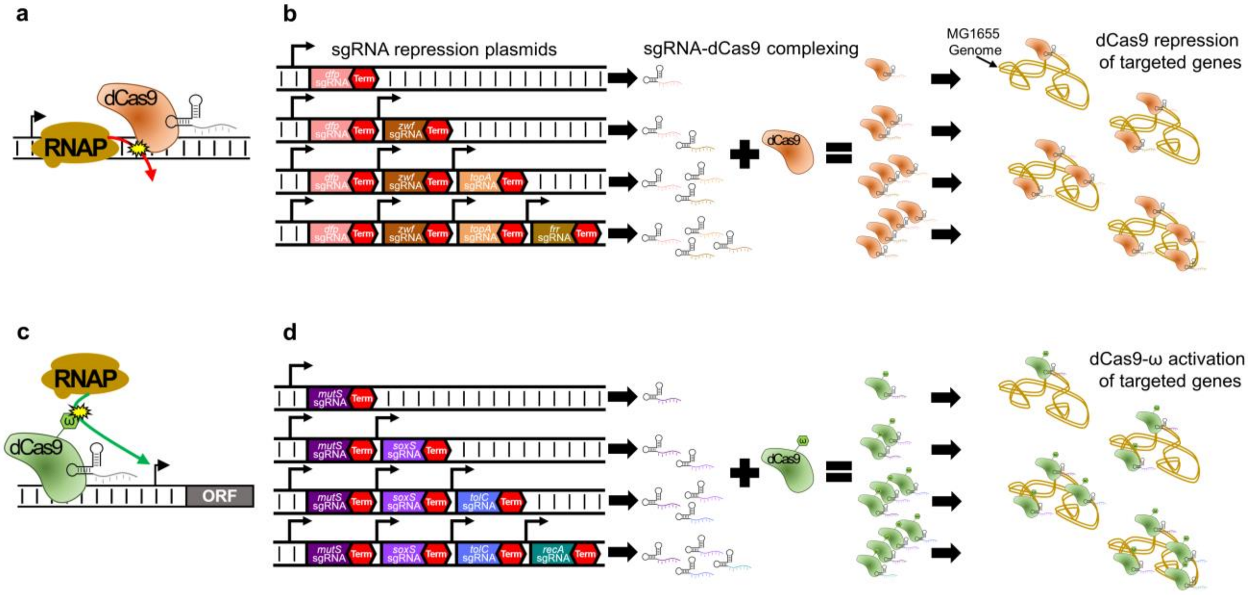

Supplementary Figure 4. Schematic depicting how dCas9 and dCas9-ω were used to perturb genes for CHAOS therapy. (**a**) dCas9 is designed to bind the +1 transcription start site of the gene targeted for expression inhibition. Upon dCas9 binding to DNA, a roadblock to transcription is created which prevents read-through of RNA polymerase (RNAP), terminating transcription and reducing mRNA production (and therefore overall gene expression). (**b**) sgRNA plasmids were created in tandem using the approach presented in Figure S5 on a separate plasmid from dCas9. These sgRNAs are independently expressed to create unique guides for particular locations of DNA. Upon complexing with dCas9, these sgRNAs guide the protein-RNA complex to unique locations on the MG1655 chromosome to inhibit expression. (**c**) dCas9-ω is designed to bind 80-100 nt upstream of the +1 promoter site for the targeted gene to activate expression. The ω-subunit of RNAP that is linked to dCas9 serves to recruit RNAP complexes to the +1 promoter site. This increases the copies of RNAP producing mRNA and thereby increases total gene expression. (**d**) In a very similar fashion as sgRNAs designed for complexing with dCas9, sgRNAs designed for complexing with dCas9-ω recruit the protein-RNA complex to unique locations for gene expression activation. As has been demonstrated in our previous work, dCas9-ω can serve in place of dCas9 to inhibit expression, and was used for inhibiting *topA*/*frr* while activating *mutS/soxS* in strain “msTf”.

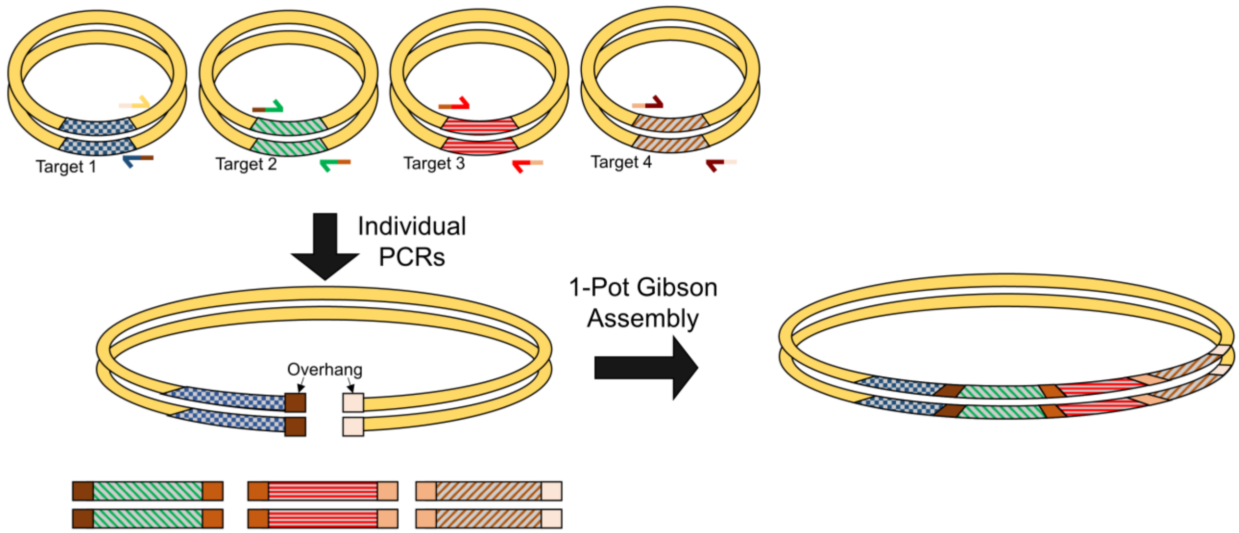

Supplementary Figure 5. Schematic depicting strategy for assembling multiple-targeting sgRNA plasmids. Individual targeting sgRNAs were first assembled, from which Gibson Assembly primers were used to amplify targets for Gibson Assembly as depicted. Sizes of final plasmids were confirmed using gel electrophoresis before transferring into the final experimental strain.

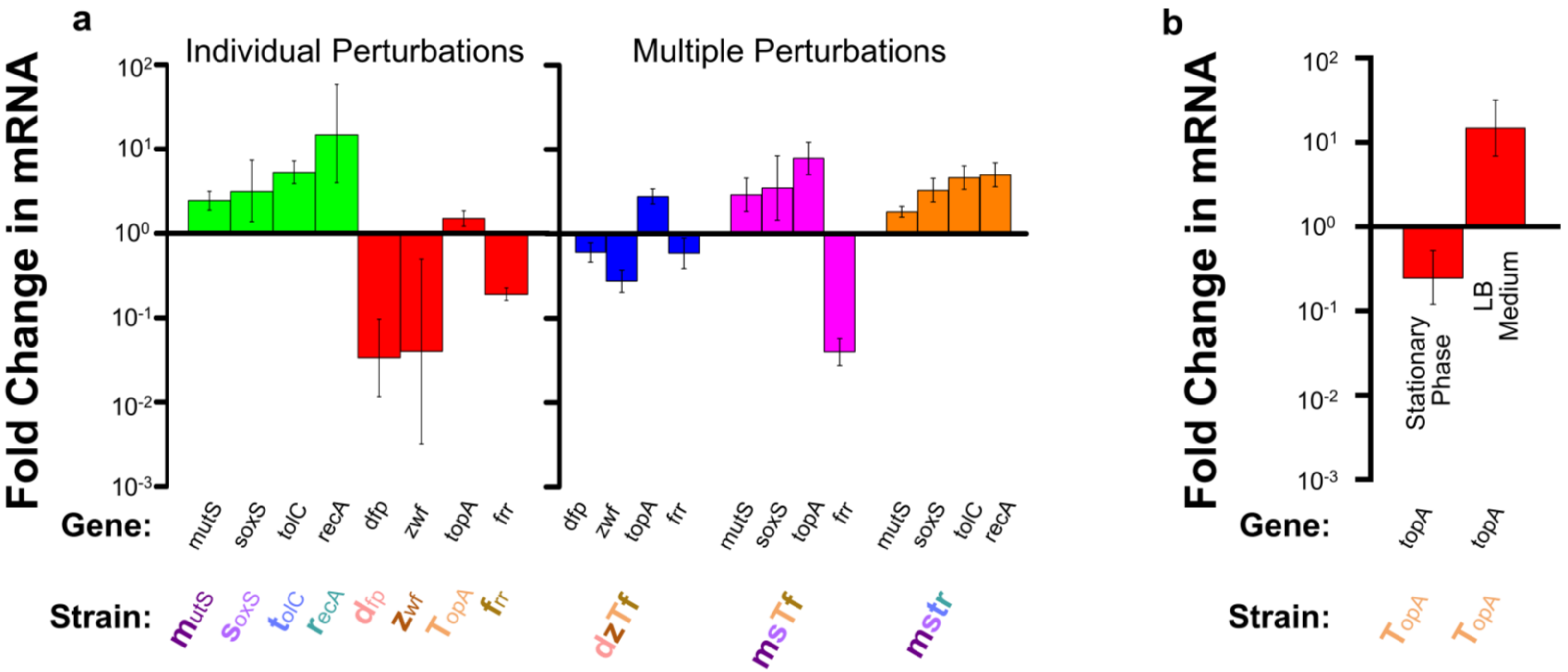

Supplementary Figure 6. RT-qPCR results of CRISPR perturbation on gene expression, as quantified by changes in mRNA concentration in relation to the single *rfp* control strain. (**a**) All qPCR was performed under 10 ng/mL aTc induction in M9 minimal media. All samples were collected after 8 hours of growth after induction during exponential phase. All error bars represent standard deviations of biological triplicates, with the average Ct of technical duplicates used for each replicate. The authors note that *recX* expression was likely increased in the strain activating *recA*, although such an increase would be substantially less than that experienced by *recA* due to a strong terminator sequence located in-between *recA* and *recX*. (**b**) We further explored the impact of CRISPR inhibition of *topA*, as this actually resulted in gene activation. We attribute this result to the inherent complex regulation of *topA*, which can be expressed from five unique promoters and is regulated by prolific binding of the phase-dependent and media-dependent transcriptional regulator, Fis. Binding of dCas9 to this promoter region, which was intended to exclude binding of RNA polymerase to the promoter closest to *topA*'s ORF, possibly instead precluded Fis binding in this region. We therefore tested *topA* expression relative to the Control strain in strains grown in both stationary phase in M9 minimal media, and in exponential phase in LB medium. We found that in stationary phase, where Fis expression is significantly lower, CRISPR inhibition worked as intended. Conversely, in LB medium, where Fis expression is significantly higher than in minimal medium, the unintended activation of *topA* by the CRISPR inhibition construct was exacerbated. These results strongly imply that the degree of *topA* perturbation by the CRISPR construct was heavily dependent on Fis concentration in the cell.

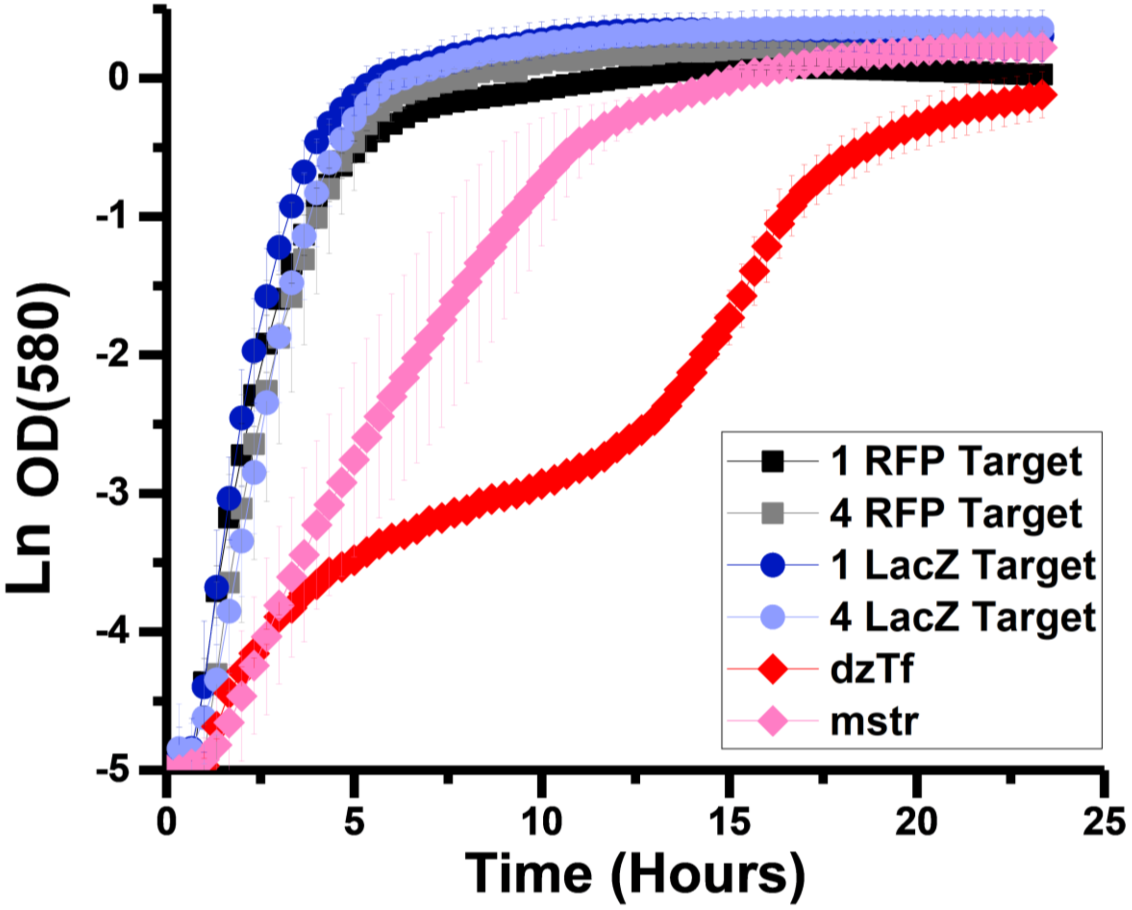

Supplementary Figure 7. Growth curves of CRISPR perturbation strains harboring one or four copies of control perturbations inhibiting expression of RFP (whose target sequence is absent from said strain), or inhibiting expression of lacZ (whose expression is not expected to affect bacterial fitness in LB medium). Also included are growth curves of the four perturbation strains targeting all four conserved genes (dzTf) or all four stress response genes (mstr) to demonstrate that selective pressure was indeed present. All growth was performed in LB medium supplemented with 10 ng/mL aTc and 0.005 µg/mL ciprofloxacin. Error bars represent standard deviation of five biological replicates.

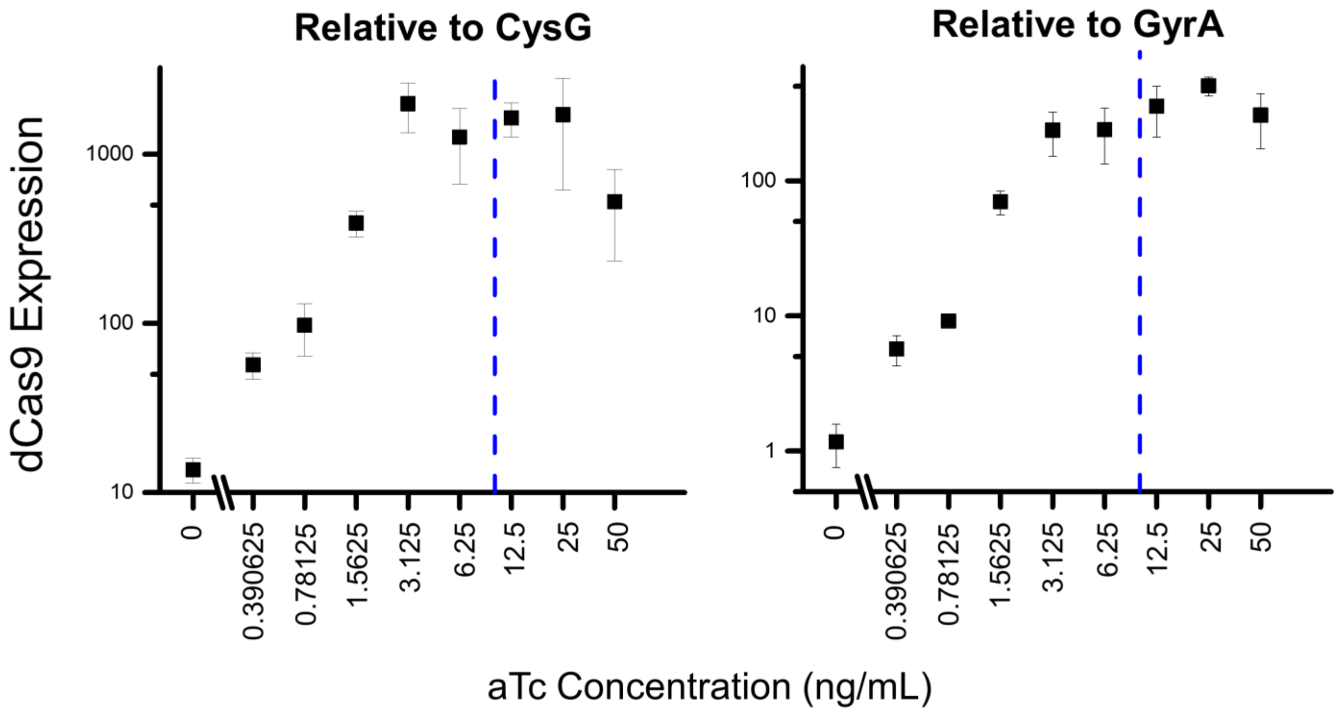

Supplementary Figure 8. Relative expression of dCas9 at various levels of aTc induction, as determined by RT-qPCR. Notably, significant leaky expression of dCas9 was observed even under no induction. Saturation of aTc induction appeared at ~3 ng/mL. The concentration of aTc used in experiments in Figs. 2-4 was 10 ng/mL and is indicated by blue dashed lines. This concentration induced ~100 fold more dCas9 expression than the basal leaky expression during inclusion of no aTc.

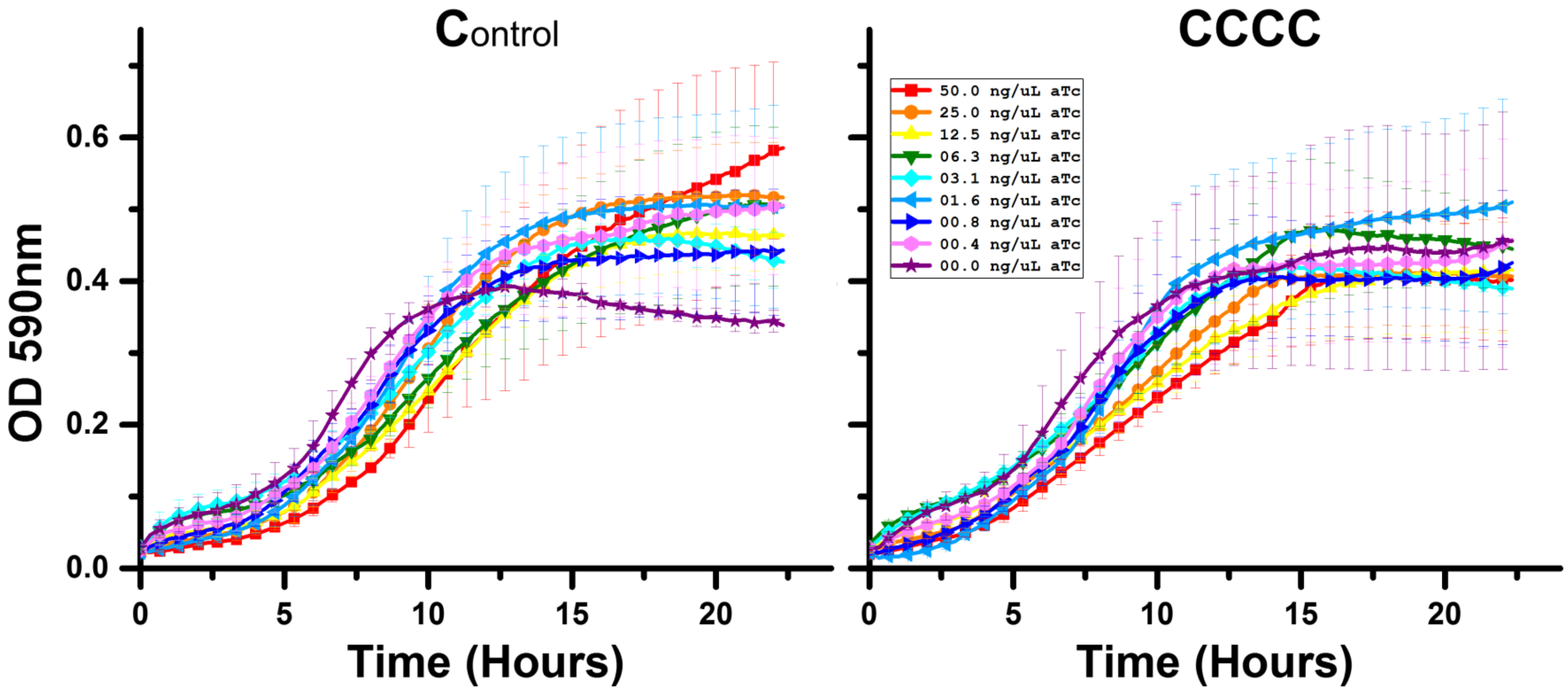

Supplementary Figure 9. Growth curves of control strains under various concentrations of aTc, representing various levels of induction of dCas9 and the CRISPR perturbation system at large. A slight growth deficit was observed from aTc toxicity correlating with higher concentrations, as would be expected for a strain of *E. coli* harboring two plasmids grown in minimal media maintaining two-antibiotic selection pressure. Error bars represent standard deviation of biological triplicates.

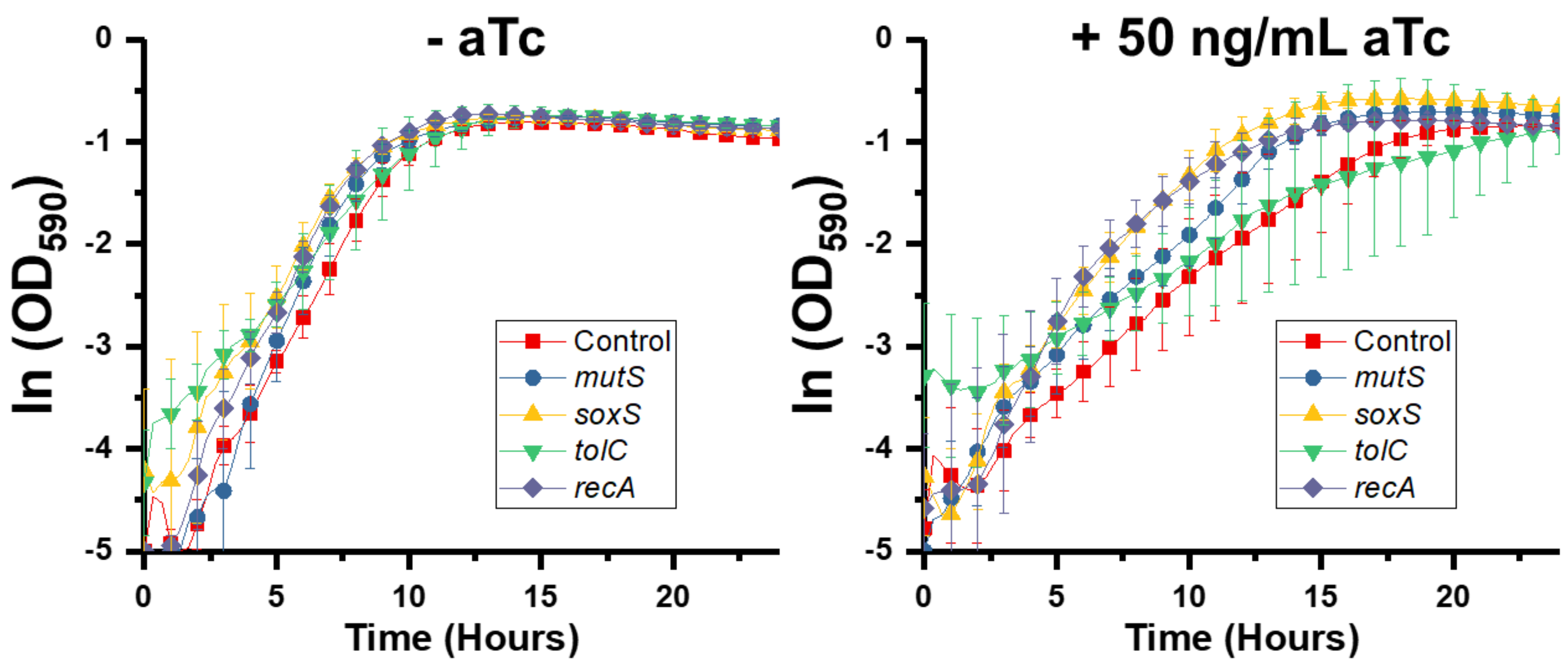

Supplementary Figure 10. Growth curves of individual perturbations of the four stress response genes with and without aTc. Optical densities are converted to logarithmic form and normalized to the starting value to highlight the exponential phase of growth.

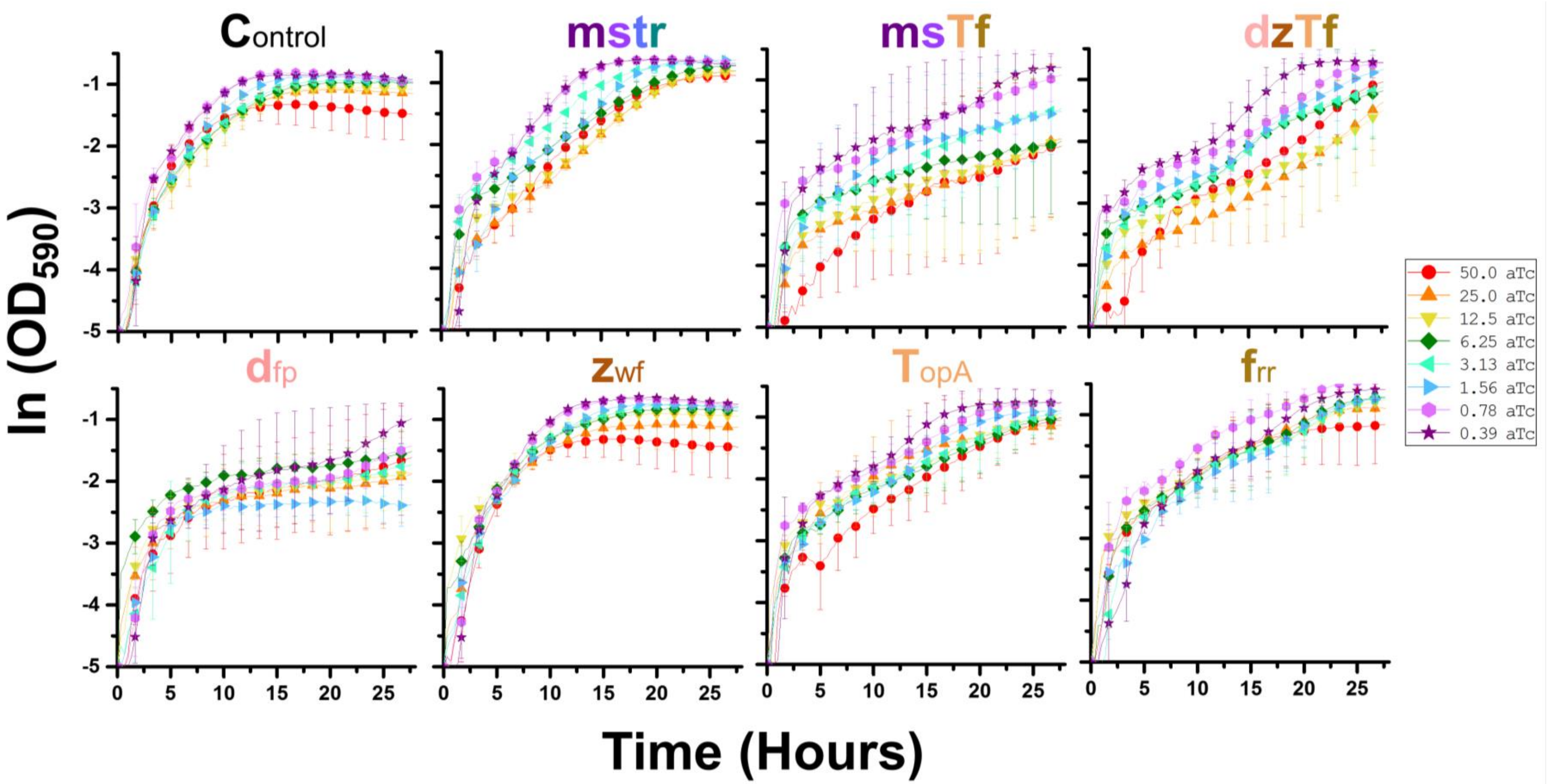

Supplementary Figure 11. Growth curves of four perturbation strains under various levels of induction of the CHAOS CRISPR perturbation system. Optical densities are converted to logarithmic form and normalized to the starting value to highlight the exponential phase of growth. The concentration of aTc used to induce expression (ng/mL) is indicated in the legend.

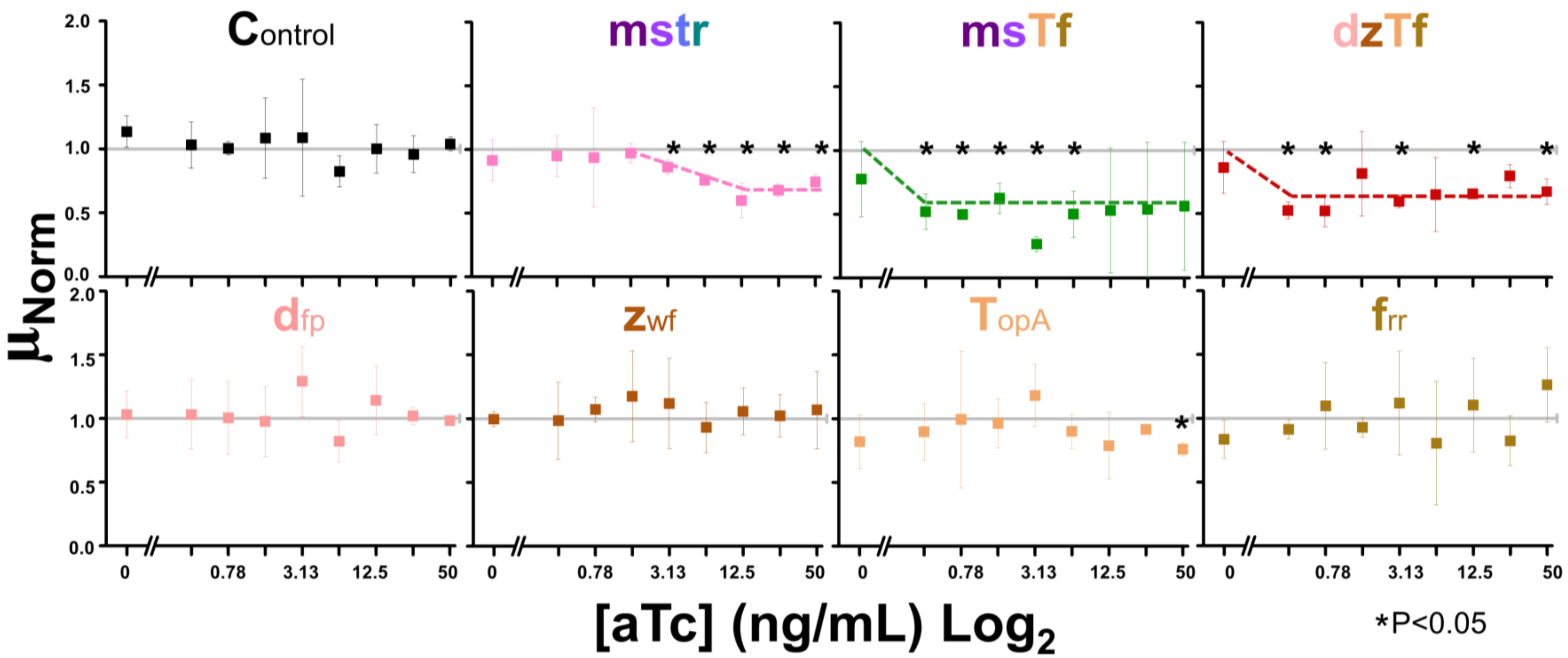

Supplementary Figure 12. Normalized growth rates of select strains under different levels of induction of the CHAOS CRISPR perturbation system. Growth rates are normalized to the single *rfp* target control strain grown under the same concentration of aTc. Dashed lines are included for the visual aid of trends. Asterisks indicate statistically significant differences in normalized growth rates from the control (P-value <0.05, two-tailed type II t-test).

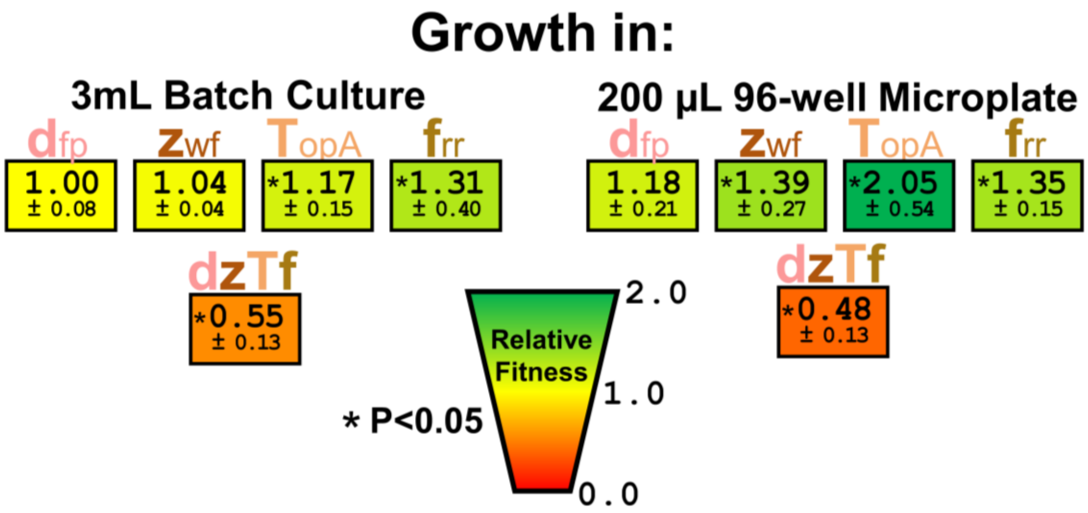

Supplementary Figure 13. Exploration of the impact of growth in microplates on experimental outcomes. The four individual perturbations of conserved genes, as well as the combined perturbations of all four simultaneously, were competed again against $C_{mCherry}$ during growth in LB medium supplemented with 10 ng/mL aTc and 0.005 µg/mL ciprofloxacin. However, growth was performed instead in 3mL LB in 5mL glass culture tubes with continuous shaking to improve culture aeration. Competitive fitness under these conditions is presented on the left, with original microplate-based fitness presented on the right. Overall, a slight reduction in the fitness benefits or detriments of perturbations appeared to emerge during growth in batch culture, likely due to an increase in cell's health in these more favorable growth conditions. However, the significant trend of lower fitness as perturbations were combined remained, suggesting that culture growth in microplates does not impact the overall finding of emergent epistasis at the gene expression level. Standard deviations were calculated from three biological triplicates for growth in 3mL batch cultures. Asterisks indicate significant differences from the eight biological replicates of strain C_{ontrol} grown in the same conditions as the right.

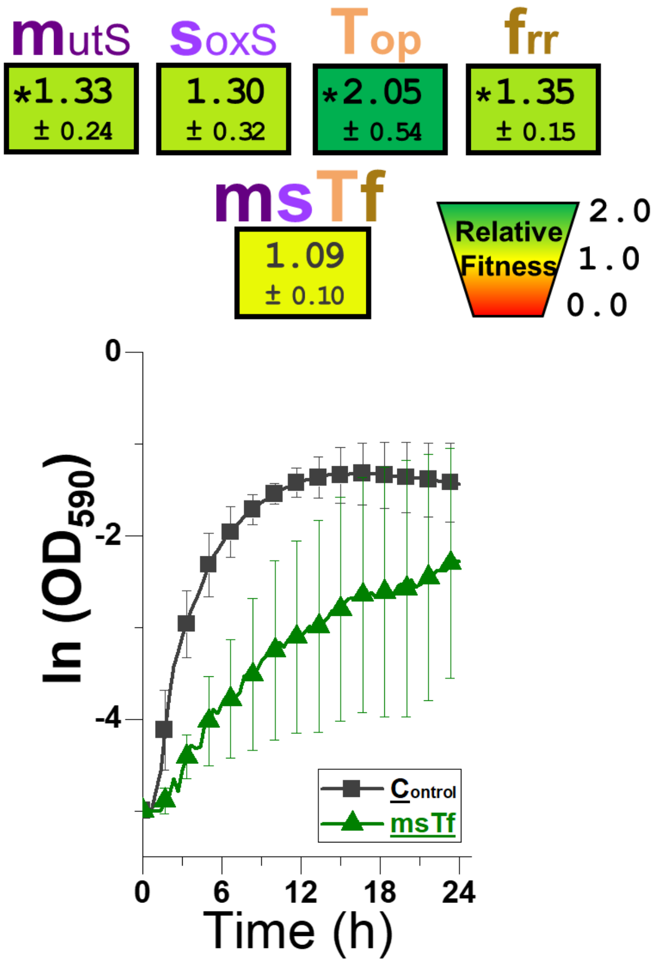

Supplementary Figure 14. Fitness of strains harboring either CRISPR activations of stress response genes (*mutS* and *soxS*) or CRISPR inhibitions of conserved genes (*topA* and *frr*) during exposure to 0.005 µg/mL ciprofloxacin and 10 ng/mL aTc in LB medium. A strain activating expression of the first two while inhibiting expression of the latter two was also constructed and tested in the same fashion. Relative fitness is listed below each strain name, followed by the standard deviation (n = 8). Asterisks indicate significant fitness differences in relation to strain "C_{ontrol}" from Figure 2 ($P < 0.01$, two-tailed type II *t*-test). Additionally, growth of strain msTf relative to the control strain was performed during exposure to 50 ng/mL aTc in M9 minimal media.

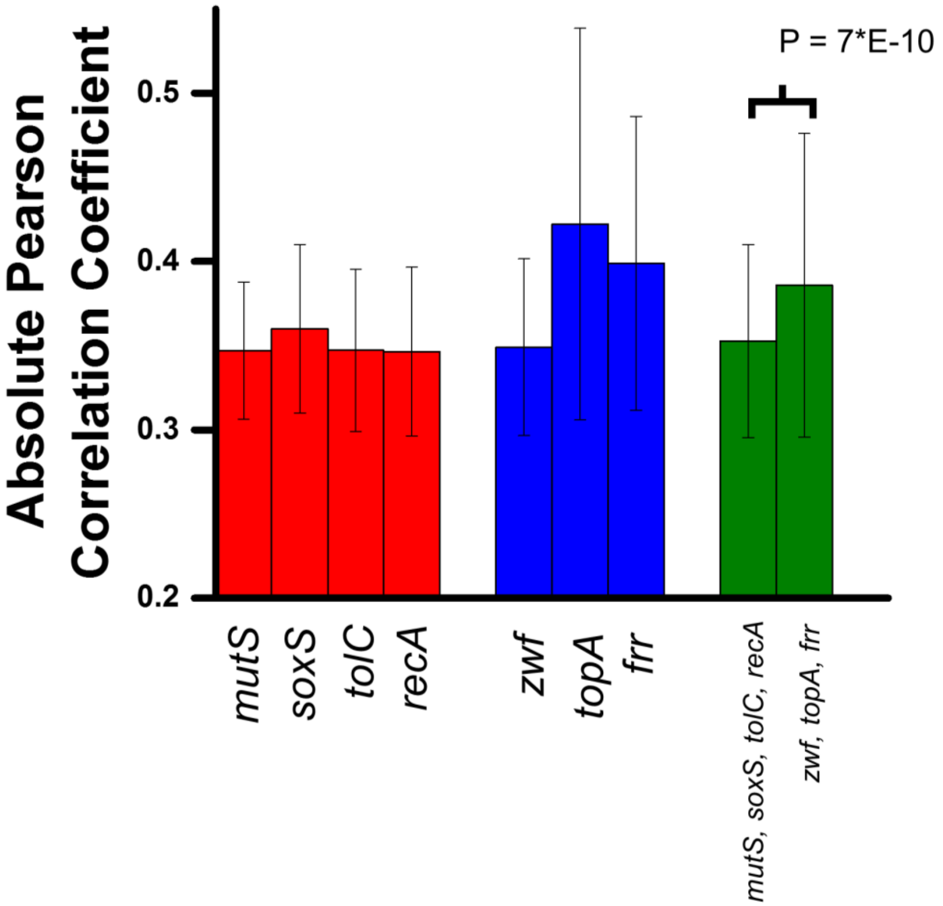

Supplementary Figure 15. Average absolute Pearson Correlation Coefficient (PCC) of statistically significant genetic interactions determined by Babu et al[2]. In this study, approximately 600,000 double-mutant strains were created from 163 gene knockouts crossed with 3,968 non-essential single gene deletions and 149 hypomorphic mutations. PCCs of phenotypes were calculated for every gene against all other genes, depicting the relative strength of that gene's genetic interaction with other genes. We took the average of the absolute value of each reported PCC of both individual genes, as well as the average within each set of genes investigated. This study did not investigate *dfp*, hence its exclusion here. We note that the average value of PCC of *mutS*, *soxS*, *tolC*, and *recA* is less than the average PCC of *zwf*, *topA*, and *frr* suggesting that the genetic interactions of the former with other genes throughout the genome are typically weaker.

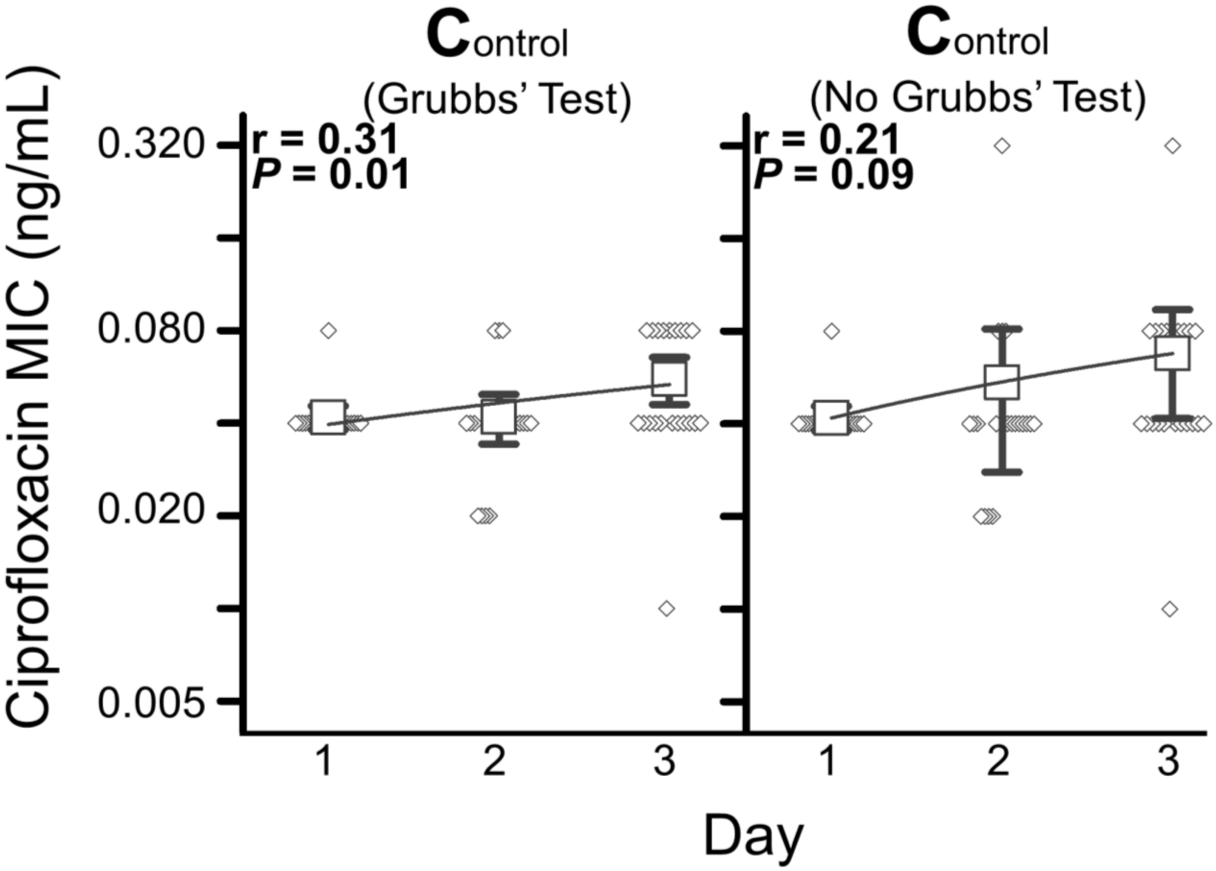

Supplementary Figure 16. Ciprofloxacin MICs of the control strain run during the same experimental run as strains dfp, zwf, topA, frr, msTf and dzTf in Figure 4. During this experimental run, one replicate of the control strain grew significantly higher than the other replicates, and sequencing of this replicate revealed a mutation resistance (see Table S3). This replicate was removed using Grubbs' test for outliers on the left and was kept in calculations on the right. The left version of the linear fit was used for all F-tests presented in Figure 4 for strains dfp, zwf, topA, frr, msTf and dzTf.

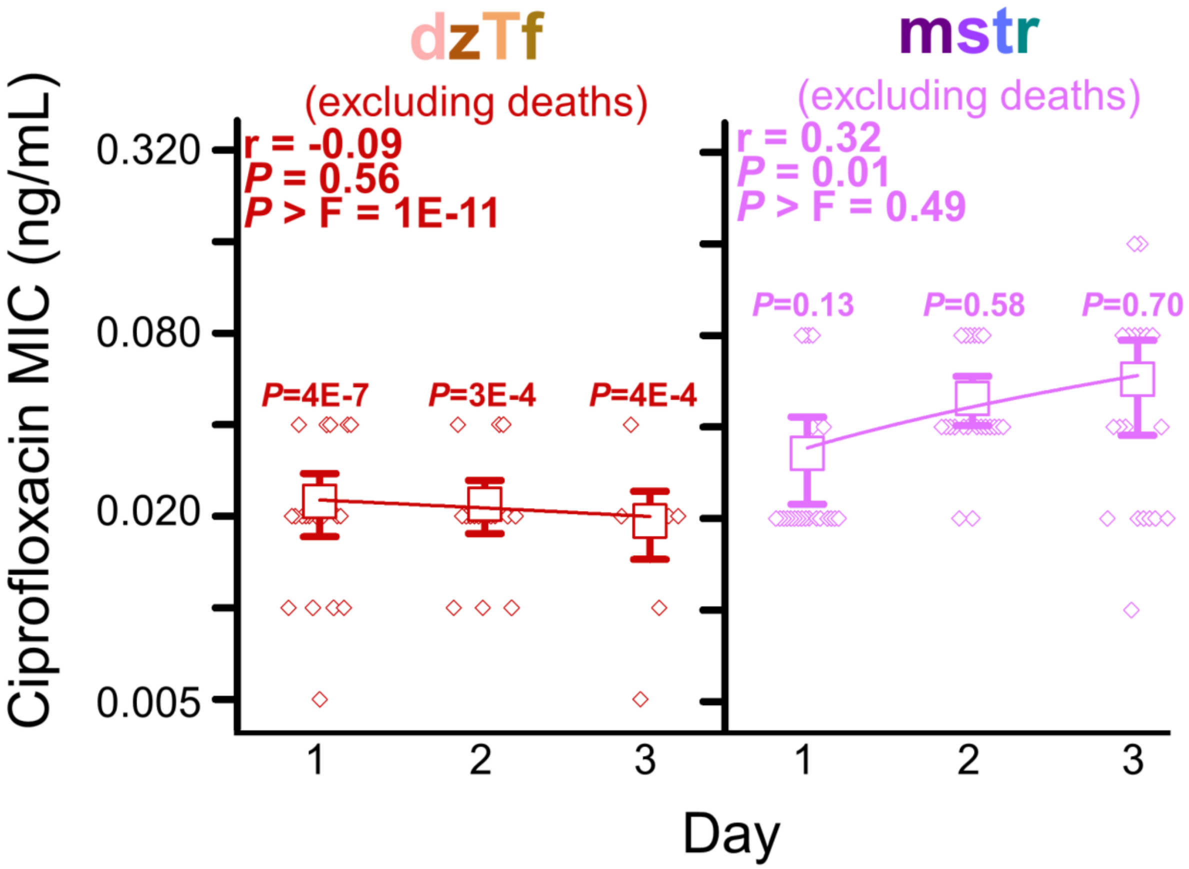

Supplementary Figure 17. Ciprofloxacin MICs from Figure 4 with slight modifications in their calculations. In Figure 4, replicates that failed to grow in absence of ciprofloxacin (i.e. replicate “deaths”) were treated as having a MIC of 0.005 µg/mL (the lowest concentration tested). In this figure, such replicates were instead removed from the dataset. This affected 3, 6, and 15 replicates on days 1, 2, and 3 respectively for strain dzTf and zero, 1, and 3 replicates on days 1, 2, and 3 respectively for strain mstr. The probability of each linear fit being different than that of the control strain during the same experimental run was calculated using F-tests, and the resulting significance is presented as *P* > F on each graph. *P* values above each data average are in relation to the control from the same day of the experiment, using a two-tailed type II t-test.

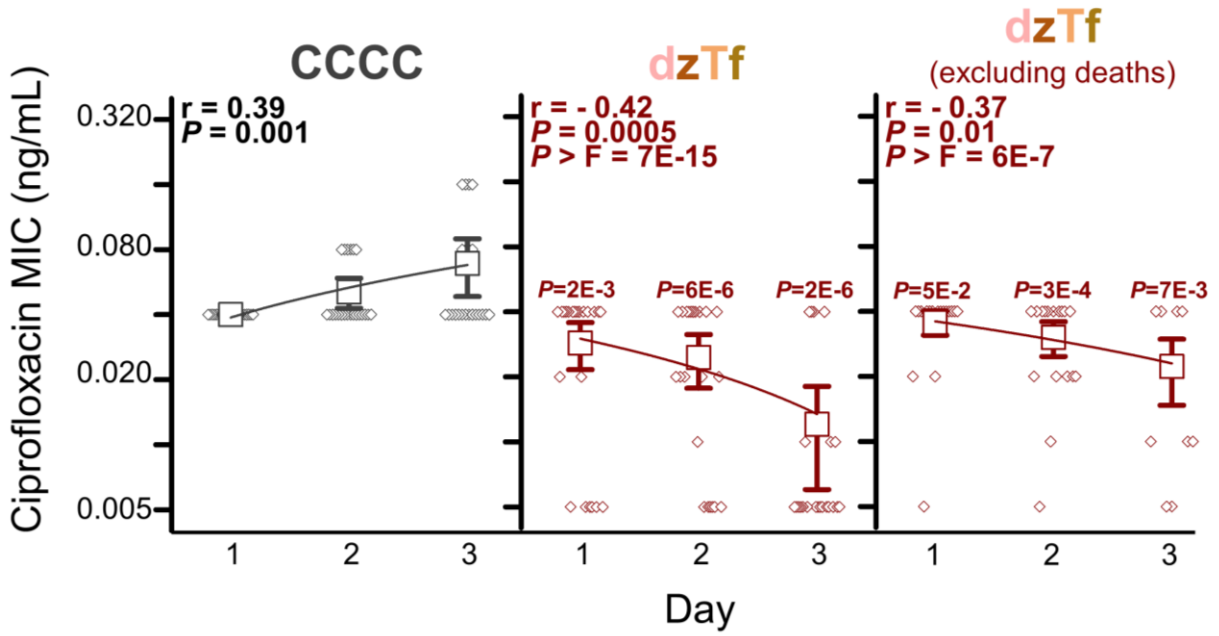

Supplementary Figure 18. Average MICs of strain dzTf compared to the control in a separate experiment to confirm the results presented in Figure 4. Replicates of dzTf that failed to grow under the absence of ciprofloxacin exposure (i.e. replicate "deaths") were treated as having a MIC of 0.005 µg/mL (the lowest concentration tested) in the middle plot or were excluded from analysis in the right plot. This affected five, five, and 13 replicates on days one, two, and three of the experiment respectively. Statistical significance of Pearson Correlation Coefficients is listed underneath their corresponding fits. The probability that the Pearson Correlation Coefficient is statistically different from the control is listed as $P > F$ on the two graphs of strain dzTf. P values above each data average are in relation to the control from the same day of the experiment, using a two-tailed type II t-test.

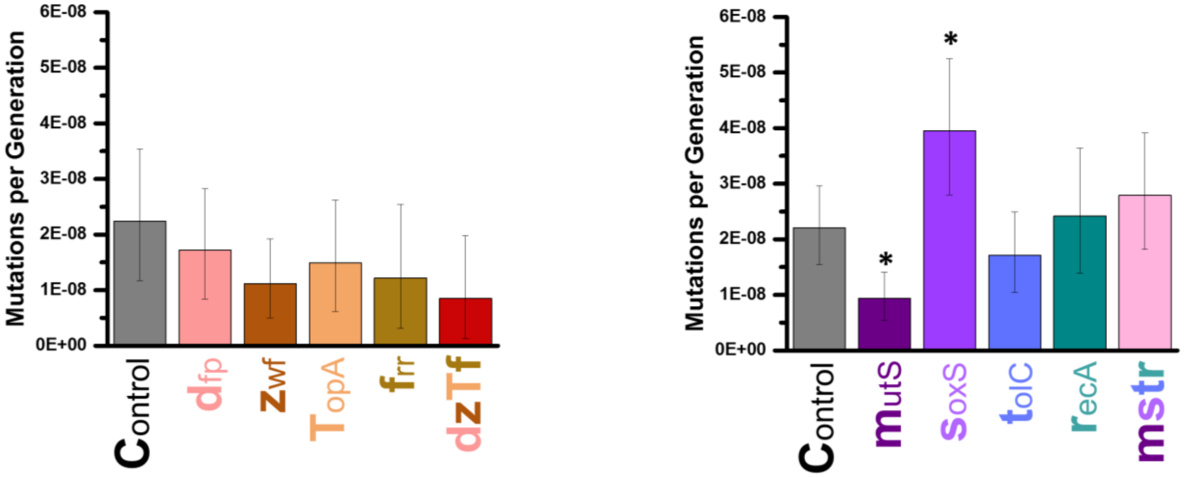

Supplementary Figure 19. Mutation rates of strains as determined by mutation fluctuation assays outlined by Luria and Delbruck. Values indicate the number of mutations per generation that arise during one day of CRISPR perturbation. All comparisons were made in relation to the control strain from the same experimental run. The FALCOR web-tool was used to calculate mutation rates and error. Error bars indicate 95% confidence interval of at least 30 biological replicates. Asterisks indicate statistically different mutation rates relative to the control strain ($P < 0.05$) calculated using a two-tailed type II t-test

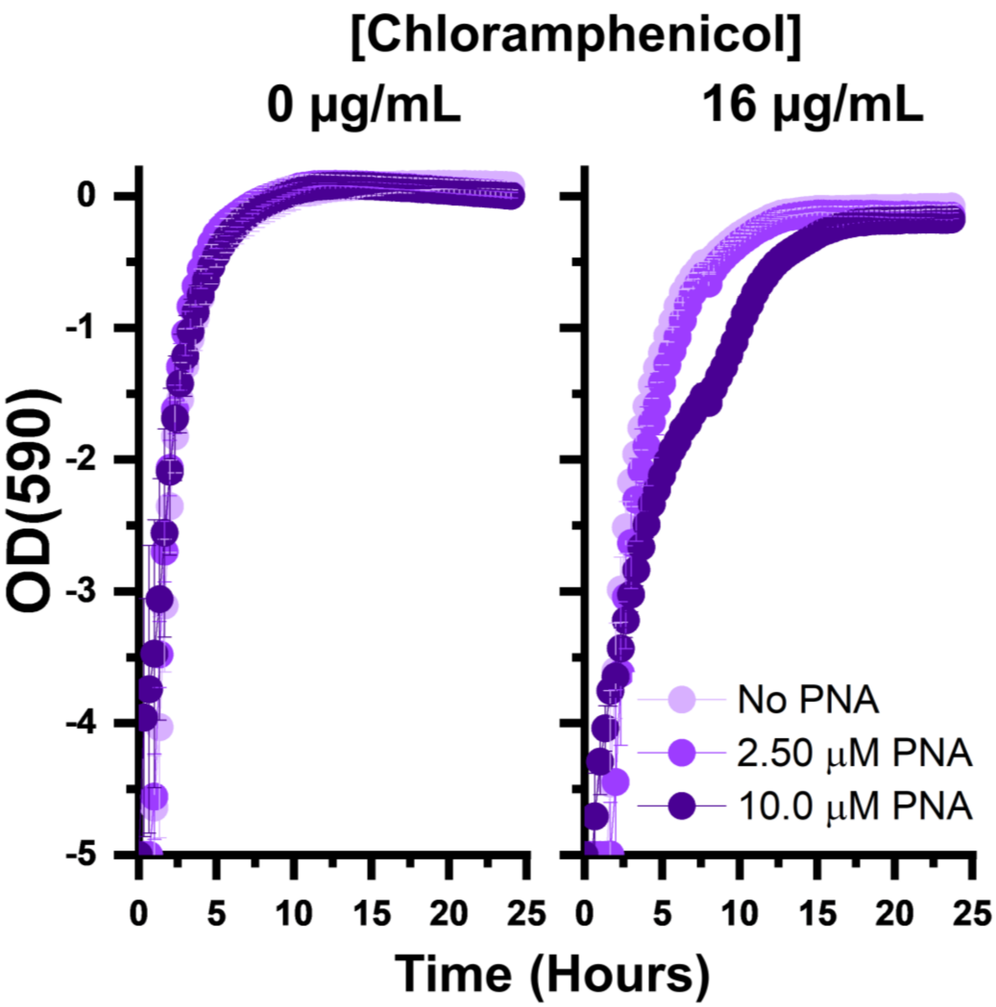

Supplementary Figure 20. Growth curves of the clinically isolated, multi-drug resistant CRE *E. coli* during exposure to various concentrations of PNA targeting a nonsense RNA sequence not present in the isolate. On the left is shown growth in the absence of chloramphenicol, and on the right is shown growth in the presence of 16 µg/mL chloramphenicol. A slight growth deficit was observed from the higher concentration of PNA only during chloramphenicol exposure. However, all samples were able to reach comparable densities by the end of 24 hours of growth, indicating that CFU values reported in Figure 6c were minimally impacted by inherently higher PNA concentrations, if at all. Error bars represent standard deviations of three biological triplicates.

Supplementary Table 1. List of 174 conserved genes across a number of bacterial species, categorized by their existence in a multi-gene operon. Genes that are transcribed individually are listed in the left column, and those that are co-transcribed with another gene are listed in the right column

Individual Transcription	
adk	murJ
alaS	nadE
argS	ndk
asd	pgsA
aspS	plsC
cdsA	priA
cysS	proC
dapB	proS
dfp	pyrH
dnaB	ribA
dnaX	ridA
efp	rluB
ffh	rplY
frr	rsmD
gltX	serS
glyA	tktA
gmk	topA
grpE	trmU
gyrA	tsaE
hisS	uppS
ispB	ydeE
metG	yoaB
metK	zwf
mnmE	

Co-Transcribed					
ackA	ftsY	lspA	rimM	rpsH	tyrS
aroK	ftsZ	map	rluD	rpsi	valS
birA	fusA	miaA	rnc	rpsJ	ybeY
cmk	gapA	mraW	rnhB	rpsL	ychF
coaD	glmS	msbA	rplB	rpsM	yebC
coaE	glmU	murC	rplC	rpsN	yejE
dapA	groL	murE	rplD	rpsO	yqgF
ddlB	groS	murG	rplF	rpsP	
def	hemC	murI	rplJ	rpsQ	
der	hemK	nrdA	rplL	rpsS	
dnaA	ileS	nusB	rplM	rutC	
dnaE	infB	obgE	rplN	secA	
dnaG	infC	pgk	rplO	secD	
dnaK	iscS	pheS	rplR	secY	
dxs	ispA	pnp	rplS	thrS	
era	ispE	ppiB	rplT	thyA	
fabD	ispG	ppnK	rplW	tmk	
fabG	lepB	prsA	rplX	trmD	
fabH	leuS	pth	rpoB	trpS	
fmt	lgt	pyrG	rpoC	trx	
folK	ligA	ribD	rpoD	tsaB	
ftsA	lolD	ribE	rpsB	tsf	
ftsI	lpd	ribF	rpsE	tufA	
ftsW	lptB	ribH	rpsF	tufB	

Supplementary Table 2. Strains used in this study. The host strain is MG1655 for all of the following. Guide RNA targets are followed by an "i" or "a" for inhibition or activation respectively.

Strain	Cas9 Phenotype	Guide RNA
**C**ontrol	dCas9-ω	*rfp*i
CCCC	dCas9-ω	*rfp*i-*rfp*i-*rfp*i-*rfp*i
C-mCherry	dCas9-ω	*rfp*i + mCherry
**m**utS	dCas9-ω	*mutS*a
**s**oxS	dCas9-ω	*soxS*a
**t**olC	dCas9-ω	*tolC*a
**r**ecA	dCas9-ω	*recA*a
ms	dCas9-ω	*mutS*a-*soxS*a
mt	dCas9-ω	*mutS*a-*tolC*a
mr	dCas9-ω	*mutS*a-*recA*a
st	dCas9-ω	*soxS*a-*tolC*a
sr	dCas9-ω	*soxS*a-*recA*a
tr	dCas9-ω	*tolC*a-*recA*a
mst	dCas9-ω	*mutS*a-*soxS*a-*tolC*a
msr	dCas9-ω	*mutS*a-*soxS*a-*recA*a
mtr	dCas9-ω	*mutS*a-*tolC*a-*recA*a
str	dCas9-ω	*soxS*a-*tolC*a-*recA*a
mstr	dCas9-ω	*mutS*a-*soxS*a-*tolC*a-*recA*a
**d**fp	dCas9	*dfp*i
**z**wf	dCas9	*zwf*i
**T**opA	dCas9	*topA*i
**f**rr	dCas9	*frr*i
dz	dCas9	*dfp*i-*zwf*i
dT	dCas9	*dfp*i-*topA*i
df	dCas9	*dfp*i-*frr*i
zT	dCas9	*zwf*i-*topA*i
zf	dCas9	*zwf*i-*frr*i
Tf	dCas9	*topA*i-*frr*i
dzT	dCas9	*dfp*i-*zwf*i-*topA*i
dzf	dCas9	*dfp*i-*zwf*i-*frr*i
dTf	dCas9	*dfp*i-*topA*i-*frr*i
zTf	dCas9	*zwf*i-*topA*i-*frr*i
dzTf	dCas9	*dfp*i-*zwf*i-*topA*i-*frr*i
msTf	dCas9-ω	*mutS*a-*soxS*a-*topA*i-*frr*i

Supplementary Table 3. Sequencing of *gyrA* after three days of ciprofloxacin adaptation.

Strain	Replicate	Day 3 MIC (µg/mL)	Mutation
C	1	0.08	N/A
			N/A
	3	0.32	Point mutation – D87Y
			Point mutation – D87Y
	4	0.08	N/A
			N/A
d	2	0.08	N/A
			N/A
	4	0.08	N/A
			N/A
	5	0.08	N/A
			N/A
z	1	0.08	N/A
			N/A
	3	0.08	N/A
			N/A
	11	0.16	N/A
			N/A
T	5	0.08	N/A
			N/A
	14	0.08	Codon deletion – S83
			Codon deletion – S83
	15	0.08	N/A
			N/A
f	1	0.08	N/A
			N/A
	4	0.08	N/A
			N/A
	20	0.16	N/A
			N/A
msTf	3	0.04	N/A
			N/A
	4	0.04	N/A
			N/A
	22	0.08	N/A
			N/A
dzTf	1	0.02	N/A
			N/A
	12	0.04	N/A
			N/A
	20	0.02	N/A
			N/A

Supplementary Table 3. Continued.

Strain	Replicate	Day 3 MIC (μg/mL)	Mutation
C	2	0.08	N/A<br>N/A
	4	0.08	N/A<br>N/A
	15	0.16	N/A<br>N/A
CCCC	2	0.08	N/A<br>N/A
	5	0.08	N/A<br>N/A
	7	0.08	N/A<br>N/A
m	3	0.08	N/A<br>N/A
	4	0.16	N/A<br>N/A
	10	0.32	N/A<br>N/A
s	5	0.08	N/A<br>N/A
	10	0.16	N/A<br>N/A
	12	0.16	Point mutation – D82G<br>Point mutation – D82G
t	6	0.16	N/A<br>N/A
	19	0.16	Codon deletion – S83<br>N/A
	20	0.16	N/A<br>N/A
r	2	0.16	N/A<br>N/A
	3	0.08	N/A<br>N/A
	12	0.16	N/A<br>N/A
mstr	3	0.08	N/A<br>N/A
	20	0.16	N/A<br>N/A
	22	0.16	N/A<br>N/A

Supplementary Table 4. A CRE isolate of *E. coli* exhibiting resistance to at least 11 antibiotics above CLSI breakpoint levels was isolated from a clinical infection (concentrations listed in μg/mL). We focused on applying CHAOS induced epistasis to re-sensitize this isolate to chloramphenicol.

	**Resistance Breakpoint**	**CRE Isolate MIC**
Ampicillin	32	>32
Ceftriaxone	4	>512
**Chloramphenicol**	**32**	**>256**
Ciprofloxacin	4	>32
Clindamycin	4	>64
Gentamicin	16	>16
Kanamycin	64	>64
Meropenem	4	>4
Nalidixic Acid	32	>32
Streptomycin	32	>256
Tetracycline	16	>16

Supplementary Table 5. Cloning, sequencing, and RT-qPCR primers used in this study. Target locations of sgRNAs are highlighted in red.

Primer Name	Sequence (5'-3')
sgRNA cloning forward – *mutS* activation	ACTAGTACTAGT**GCAAGTACGCAAAATTGTAT**GTTTTAGAGCTAGAAATAGC
sgRNA cloning forward – *soxS* activation	ACTAGTACTAGT**GCGTTTCGCCACTTCGCCGG**GTTTTAGAGCTAGAAATAGC
sgRNA cloning forward – *tolC* activation	ACTAGTACTAGT**AGCAGTCATGTGTTAAATTG**GTTTTAGAGCTAGAAATAGC
sgRNA cloning forward – *recA* activation	ACTAGTACTAGT**CCGTGATGCGGTGCGTCGTC**GTTTTAGAGCTAGAAATAGC
sgRNA cloning forward – *dfp* inhibition	ACTAGTACTAGT**GTGATAAAATCGCCAACTTC**GTTTTAGAGCTAGAAATAGC
sgRNA cloning forward – *zwf* inhibition	ACTAGTACTAGT**GTATACTTGTAATTTTCTTA**GTTTTAGAGCTAGAAATAGC
sgRNA cloning forward – *topA* inhibition	ACTAGTACTAGT**CTGGCAACGAGTTACCGATA**GTTTTAGAGCTAGAAATAGC
sgRNA cloning forward – *frr* inhibition	ACTAGTACTAGT**AGCCCTGATTAAACATATTA**GTTTTAGAGCTAGAAATAGC
sgRNA cloning single target reverse	GGGCCCGGGCCCAAGCTTCAAAAAAAGCACCG
sgRNA plasmid sequencing	GGGGGGGACGTCTAAGAAACCATTATTATCATG
Gibson primer – backbone Forward	CTCAGGTTCATCGTTTGCATGGGATCACAGGTCTAAGAAACCATTATTATCATGACATTA
Gibson primer – backbone Reverse	ATTGACAGCTGAGAGCGCCTTCACAACGTGGTCAGGTGGCACTTTTCGGGGAAATGTGCG
Gibson primer – insert 1 Forward	CACGTTGTGAAGGCGCTCTCAGCTGTCAATCTCGAGTAAGGATCCAGTTCACCGACAAAC
Gibson primer – insert 1 Reverse	CTGTGATCCCATGCAAACGATGAACCTGAGAATTCTAAAGATCTTTGACAGCTAGCTCAG
Gibson primer – insert 2 Forward	TCACCAAATTATAGCCATACAGACCCAAATCTCGAGTAAGGATCCAGTTCACCGACAAAC
Gibson primer – insert 2 Reverse	ATTTGGGTCTGTATGGCTATAATTTGGTGAAATTCTAAAGATCTTTGACAGCTAGCTCAG
Gibson primer – insert 3 Forward	TTTAAATCATATCACACAATTAGCCTCTCGCTCGAGTAAGGATCCAGTTCACCGACAAAC
Gibson primer – insert 3 Reverse	CGAGAGGCTAATTGTGTGATATGATTTAAAAATTCTAAAGATCTTTGACAGCTAGCTCAG
Cloning of mCherry from plasmid PHL662 to sgRNA plasmid, Forward	GACGTCGACGTCTAAGAAACCATTATTATCAT
Cloning of mCherry from plasmid PHL662 to sgRNA plasmid, Reverse	GACGTCGACGTCTTACTTGTACAGCTCGTCCA
gyrA sequencing amplification Forward	GCCTTCCACGCGTTTTTCTT
gyrA sequencing amplification Reverse	AACCGACATCGAGCACCTTT
qPCR *mutS* Forward	ATGGAACGTGAGCAGGACAG
qPCR *mutS* Reverse	CAGCCAGCGTTTCAGCATAC
qPCR *soxS* Forward	TCTGCTGCGAGACATAACCC
qPCR *soxS* Reverse	ACTTGCAACGAATGTTCCGC
qPCR *tolC* Forward	ACGCACTACCACCAGTAACG
qPCR *tolC* Reverse	TTTGTCTTCCGGGACCAGTG
qPCR *recA* Forward	ATCGCCTGGCTCATCATACG
qPCR *recA* Reverse	GCACTGGAAATCTGTGACGC
qPCR *dfp* Forward	TTTGATTGCCCGTGTTGCTG
qPCR *dfp* Reverse	CGGGAAGCAAGCACCTCTAA
qPCR *zwf* Forward	ACGAAGTGGAAGAAGCCTGG
qPCR *zwf* Reverse	TCACGGGTAATCATCGCCAC
qPCR *topA* Forward	CTTGTGGTCGCAAAATGGGG
qPCR *topA* Reverse	GCACGTTCAGCACTTCGTTT
qPCR *frr* Forward	AAGCGATTATGGCGTCCGAT
qPCR *frr* Reverse	GCTTGTTCTGCTTCACCACG
qPCR *gyrA* Forward	GTCATAGACCGCCGAGTCAC
qPCR *gyrA* Reverse & *gyrA* sequencing	GCGATGTCGGTCATTGTTGG
qPCR *cysG* Forward	ATTCCGTTCTCGGTGGTTCC
qPCR *cysG* Reverse	CCAGCGTCTGTTTTTCTGCC
qPCR *dCas9* Forward	AGATTCGCAAACGCCCTCTA
qPCR *dCas9* Reverse	CTCCTTGGAGAATCCGCCTG

SUPPLEMENTARY REFERENCES

1. Szklarczyk, D. *et al.* STRING v10: Protein-protein interaction networks, integrated over the tree of life. *Nucleic Acids Res.* **43,** D447–D452 (2015).

2. Babu, M. *et al.* Quantitative Genome-Wide Genetic Interaction Screens Reveal Global Epistatic Relationships of Protein Complexes in Escherichia coli. *PLoS Genet.* **10,** (2014).
