# Supplementary material for: Multiplexed deactivated CRISPR-Cas9 gene expression perturbations deter bacterial adaptation by inducing negative epistasis
Source: Commun Biol. 2018 Sep 3;1:129. doi: 10.1038/s42003-018-0135-2 (PMC6123780; doi:10.1038/s42003-018-0135-2)
Supplement: Supplementary file 2 — Description of Additional Supplementary Files [file 42003_2018_135_MOESM2_ESM.docx]

**Description of Additional Supplementary Files**

File Name: Supplementary Data 1

Description: Tabulated calculations of fitness, epistasis, and minimum inhibitory concentrations. In the first excel tab, raw epistasis calculations used in figure 3 are presented along with associated significance. In the second and third tabs, the maximum concentration of ciprofloxacin (in µg * mL-1) to which each replicate survived is presented. These values were used to construct Figure 3. In the final tab four, the maximum concentration of ciprofloxacin (in µg * mL-1) survived is presented for a repeated experiment of strain dzTf and the control strain.
